# Efficient Multiplex Genome Editing in *Streptomyces* via Engineered CRISPR-Cas12a Systems

**DOI:** 10.3389/fbioe.2020.00726

**Published:** 2020-06-30

**Authors:** Jun Zhang, Dan Zhang, Jie Zhu, Huayi Liu, Shufang Liang, Yunzi Luo

**Affiliations:** ^1^Department of Gastroenterology, State Key Laboratory of Biotherapy, West China Hospital, Sichuan University and Collaborative Innovation Center of Biotherapy, Chengdu, China; ^2^State Key Laboratory of Biotherapy and Cancer Center, West China Hospital, Sichuan University and Collaborative Innovation Center of Biotherapy, Chengdu, China; ^3^Key Laboratory of Systems Bioengineering (Ministry of Education), Frontiers Science Center of Synthetic Biology, School of Chemical Engineering and Technology, Tianjin University, Tianjin, China

**Keywords:** CRISPR, *Fn*Cas12a, *Streptomyces*, genome editing, PAM recognition

## Abstract

*Streptomyces* strains produce a great number of valuable natural products. With the development of genome sequencing, a vast number of biosynthetic gene clusters with high potential for use in the discovery of valuable clinical drugs have been revealed. Therefore, emerging needs for tools to manipulate these biosynthetic pathways are presented. Although the clustered regularly interspaced short palindromic repeats/CRISPR-associated protein 9 (CRISPR/Cas 9) system has exhibited great capabilities for gene editing in multiple *Streptomyces* strains, it has failed to work in some newly discovered strains and some important industrial strains. Additionally, the protospacer adjacent motif (PAM) recognition scope of this system sometimes limits its applications for generating precise site mutations and insertions. Here, we developed three efficient CRISPR-*Fn*Cas12a systems for multiplex genome editing in several *Streptomyces* strains. Each system exhibited advantages for different applications. The CRISPR-*Fn*Cas12a1 system was efficiently applied in the industrial strain *Streptomyces hygroscopicus*, in which *Sp*Cas9 does not work well. The CRISPR-*Fn*Cas12a2 system was used to delete large fragments ranging from 21.4 to 128 kb. Additionally, the CRISPR-*Fn*Cas12a3 system employing the engineered *Fn*Cas12a mutant EP16, which recognizes a broad spectrum of PAM sequences, was used to precisely perform site mutations and insertions. The CRISPR-*Fn*Cas12a3 system addressed the limitation of TTN PAM recognition in *Streptomyces* strains with high GC contents. In summary, all the CRISPR-*Fn*Cas12a systems developed in this study are powerful tools for precise and multiplex genome editing in *Streptomyces* strains.

## Introduction

*Streptomyces*, the largest genus of actinobacteria, has been well studied, as it contains the most prolific producers of a vast array of bioactive natural products, including antibiotics, antifungals, and anticancer agents (Baltz, 2008; Zhu et al., 2011; Cho et al., 2017; Frattaruolo et al., 2017). Over the past decades, large-scale genome sequencing efforts have revealed that great potential still remains for the discovery of new natural products produced by members of this genus. Thus, increasing numbers of genetic engineering tools have been developed to explore these products (Luo et al., 2015b, 2016; Zhao et al., 2019). In particular, with the rapid development of the clustered regularly interspaced short palindromic repeats (CRISPR)/CRISPR-associated protein (Cas) system (Jiang et al., 2013; Mali et al., 2013; Yan et al., 2018), effective genome editing has become increasingly easy and convenient, paving the way for us to assemble or activate uncharacterized gene clusters. However, CRISPR/Cas9 has been reported to not work in some *Streptomyces* strains, such as *Streptomyces* sp. KY 40-1 (Salem et al., 2017), *Streptomyces* sp. NRRL S-244 (Yeo et al., 2019) and *Streptomyces hygroscopicus* SIPI-KF (Li et al., 2018), because of its toxicity to the hosts.

Cas12a, a Class 2 CRISPR effector, is a single RNA-guided endonuclease (Zetsche et al., 2015). Due to their simplicity, *As*Cas12a from *Acidaminococcus* sp. BV3L6 and *Lb*Cas12a from *Lachnospiraceae bacterium* ND2006 have generally been applied to mammalian cells (Toth et al., 2016) and plants (Tang et al., 2017), while *Fn*Cas12a from *Francisella novicida* U112 has successfully been applied to yeast (Swiat et al., 2017), *Corynebacterium glutamicum* (Jiang et al., 2017) and other bacteria (Ungerer and Pakrasi, 2016). Wild-type (WT) *Fn*Cas12a has been used in *Streptomyces*, particularly in some strains in which the CRISPR/Cas9 system does not function properly, such as *S. hygroscopicus* SIPI-KF (Li et al., 2018) and *Streptomyces* sp. NRRL S-244 (Yeo et al., 2019).

However, the TTN protospacer adjacent motif (PAM) requirement restricts the application of *Fn*Cas12a in *Streptomyces* strains, which are GC rich. In particular, target site selection is limited for site mutations or insertions in the genome. Thus, there remains a demand for highly efficient and versatile genome editing tools for *Streptomyces.* Recently, we constructed an engineered *Fn*Cas12a variant, EP16, which shows broad PAM recognition abilities *in vitro*, including YN (Y = C or T), TAC and CAA (Wang et al., 2019). Therefore, EP16 provides additional opportunities to precisely edit the high-GC-contents genomes of *Streptomyces* strains.

**FIGURE 1:**
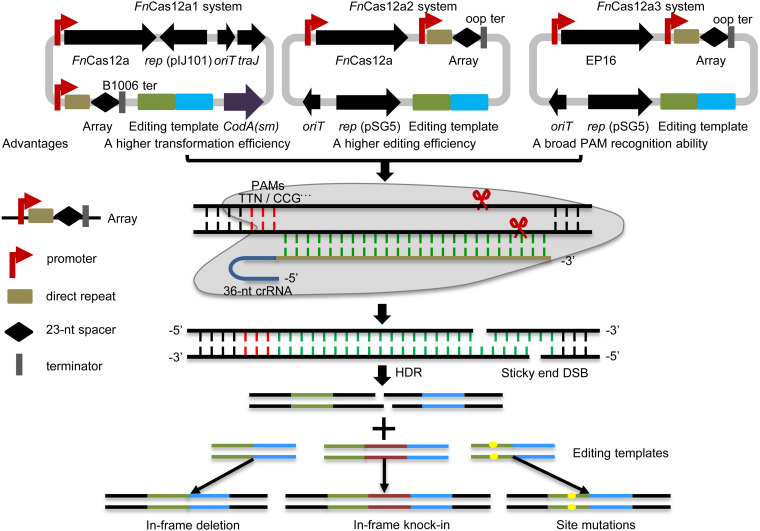
Overview of the *Fn*Cas12a systems. Three *Fn*Cas12a systems were developed. The *Fn*Cas12a1 system carries a crRNA terminated by the B1006 terminator, *Fn*Cas12a, *codA(sm)*, *traJ*, *oriT*, and the pIJ101 replicon. The *Fn*Cas12a2 system carries a crRNA terminated by the oop terminator, *Fn*Cas12a, and partial sequences of *traJ*, oriT, and the pSG5 replicon. The *Fn*Cas12a3 system contains the same elements as the *Fn*Cas12a2 system, except that it contains the *Fn*Cas12a mutant EP16. *Fn*Cas12a1 and *Fn*Cas12a2 cleave DNA with TTN PAM recognition sites, while the *Fn*Cas12a3 system targets the YN (Y = C or T), TAC and CAA PAM recognition sites. These systems were applied to generate gene deletions, site mutations and insertions via HDR in *Streptomyces* strains. DSB, double-strand break.

In this study, three optimized CRISPR-*Fn*Cas12a systems, CRISPR-*Fn*Cas12a1, CRISPR-*Fn*Cas12a2 and CRISPR-*Fn*Cas12a3 (Figure 1 and Supplementary Table 2), were developed to edit the genomes of different *Streptomyces* strains based on homology-directed repair (HDR), as non-homologous end joining (NHEJ) does not occur in most streptomycetes. (i) The CRISPR-*Fn*Cas12a1 system exhibited a higher transformation efficiency than the CRISPR-*Fn*Cas12a2 system, as it contains a *traJ* gene encoding an activator of the transfer operon (Will and Frost, 2006). (ii) A higher editing efficiency was observed for the CRISPR-*Fn*Cas12a2 system than the CRISPR-*Fn*Cas12a1 system when *Fn*Cas12a was driven by the constitutive promoters *kasO*p^∗^ and rpsLp(XC). (iii) The *Fn*Cas12a3 system containing the *Fn*Cas12a mutant EP16 (N607R/K613V/N617R/K180S/K660R/D616N) with expanded PAM recognition ability was used to generate site mutation, insertion and subsequent biosynthetic gene cluster activation (Figure 1). Overall, our findings describe powerful tools for precise genome editing and subsequently for discovering and activating valuable natural products in *Streptomyces* strains.

## Materials and Methods

### Strains, Cultivation and Reagents

*Streptomyces coelicolor* A3(2), *Streptomyces griseus*, *Streptomyces lividans*, *E. coli* DH5α and *E. coli* ET12567/pUZ8002 were gifts from Professor Huimin Zhao at the University of Illinois at Urbana-Champaign. *S. hygroscopicus* NRRL5491 and *Streptomyces roseosporus* NRRL 11379 were purchased from the American Type Culture Collection (ATCC, Manassas, VA, United States). All *Streptomyces* strains were grown in MYG liquid medium (10 g/L malt extract broth, 4 g/L yeast extract, and 4 g/L glucose) or on M-ISP4 agar medium (1 g/L yeast extract, 2 g/L tryptone, 5 g/L soluble starch, 5 g/L D-mannitol, 5 g/L soya flour, 1 g/L NaCl, 2 g/L (NH_4_)_2_SO_4_, 1 g/L K_2_HPO_3_, 2 g/L CaCO_3_, 20 g/L agar, 1 g/L FeSO_4_, 1 g/L MnCl_2_, and 1 g/L ZnSO_4_, pH 7.0) at 30°C. Next, 2 × YT broth (1% yeast extract, 1% tryptone, and 0.5% NaCl, pH 7.0) was used for strain washing and spore germination before conjugation. M-ISP4 agar medium supplemented with 25 mM MgCl_2_ or 20 μg/mL apramycin and 40 μg/mL nalidixic acid was used for conjugation from *E. coli* ET12567/pUZ8002 to *Streptomyces* (Kieser et al., 2000) or for plasmid selection in *Streptomyces.* R2YE medium supplemented with 20 μg/mL apramycin and 40 μg/mL nalidixic acid was used to screen potentially edited *Streptomyces. E. coli* DH5α was grown in Luria-Bertani (LB) broth with 50 μg/mL apramycin and used for plasmid cloning and maintenance. *E. coli* ET12567/pUZ8002 was grown in LB broth supplemented with 25 μg/mL apramycin, 12.5 μg/mL chloramphenicol and 25 μg/mL kanamycin.

D-Mannitol was obtained from Sigma-Aldrich (St. Louis, MO, United States). All media components of LB broth were purchased from Oxoid (Basingstoke, Hampshire, United Kingdom), and other reagents added to media were purchased from Sangon Biotech Co., Ltd. (Shanghai, China). PCR was performed with Q5 DNA polymerase (New England Biolabs, Ipswich, MA, United States) or T5 mix (Tsingke, Beijing, China). All PCR products were purified using a Wizard Genomic DNA Purification Kit (Promega, Madison, WI, United States). The restriction endonucleases *Xba*I, *Bbs*I, and CIP were purchased from New England Biolabs. A one-step cloning kit for two or more fragment assemblies was purchased from Vazyme Biotech Co., Ltd. (Nanjing, Jiangsu, China).

### Plasmid Construction

Three CRISPR-*Fn*Cas12a systems (CRISPR-*Fn*Cas12a1, CRISPR-*Fn*Cas12a2 and CRISPR-*Fn*Cas12a3) were constructed in this study. The CRISPR-*Fn*Cas12a1 system was constructed based on pWHU2653 (Zeng et al., 2015). The CRISPR-*Fn*Cas12a2 system and the CRISPR-*Fn*Cas12a3 system were constructed based on pCRISPomyces-2 (Cobb et al., 2015). The CRISPR-*Fn*Cas12a1 system consisted of a counterselection marker *codA(sm)*. The CRISPR-*Fn*Cas12a systems were constructed in several steps. The *kasO*p^∗^ promoter (Wang et al., 2013), rpsLp(XC) promoter (Shao et al., 2013), *ermE*p^∗^ promoter (Luo et al., 2015a), and Potr^∗^ system (Wang et al., 2016) were amplified by PCR from template plasmids. *Streptomyces* codon-optimized *Fn*Cas12a was chemically synthesized by Genewiz (Suzhou, Jiangsu, China). Other elements, including the *lacZ* cassette, unique *Bbs*I and *Xba*I restriction sites, temperature-sensitive pSG5 *rep* region, *aac(3)IV* coding sequence, *colE1* origin, and transfer *oriT* region, were amplified from the pWHU2653 or pCRISPomyces-2 plasmid. The yeast helper fragment was amplified from pRS416, which was a gift from Professor Huimin Zhao. All fragments were assembled into a plasmid with a one-step assembly kit or DNA assembler (Shao et al., 2009). A specific 23-nt spacer with a TTN PAM sequence located at the 5′ end of the coding strand was chosen. The PAM sequence together with the nearby 12-nt spacer sequence was analyzed using BLAST to confirm its specificity. The 19-nt or 36-nt direct repeat (DR) sequences and the 23-nt spacer targeting genes and gene clusters were synthesized and inserted into pYL-*Fn*Cas12a plasmids through Golden Gate assembly. Next, two 1- or 2-kb homologous arms corresponding to the upstream and downstream regions of the target genes or gene clusters were amplified from purified genomic DNA and fused into the *Xba*I site of the desired plasmid by one-step assembly. The presence of the correct plasmids was confirmed by sequencing (Tsingke, Beijing, China). All primers used in this study are listed in Supplementary Table 1.

### Transformation

DNA was transformed into *E. coli* DH5α or ET12567/pUZ8002 using the heat shock method. Yeast transformation was performed by electroporation following a protocol described in a previous study (Shao et al., 2009). The conjugation of plasmids from *E. coli* ET12567/pUZ8002 to *Streptomyces* strains was performed following a previously described protocol (Kieser et al., 2000).

### Screening of Potentially Edited Streptomyces Strains

Seven days after conjugation, seven single exconjugants were randomly picked, restreaked on R2YE or M-ISP4 agar plates supplemented with 20 μg/mL apramycin and 40 μg/mL nalidixic acid, and grown at 30°C for 7 days. Then, mycelia were collected from the plates for genomic DNA isolation using a bacterial genomic DNA extraction kit (Tiangen, Beijing, China). Deletions were identified using PCR amplification of the genomic DNA or spores with primers located upstream and downstream of the target genes or primers annealing within or outside of the target gene clusters. Then, sequencing of the PCR products was performed to confirm the sequences (Tsingke, Beijing, China). For CRISPR-*Fn*Cas12a1 system clearance, M-ISP4 plates containing 800 μg/mL 5FC were used to cultivate the strains in the dark at 28°C for 3 or 4 days. For the CRISPR-*Fn*Cas12a2 system, plasmid clearance was carried out through high-temperature cultivation at 37°C for 2–3 days after normal cultivation at 30°C for 1 day.

### Transcription Analysis of *Fn*Cas12a by Real-Time PCR (RT-qPCR)

Spores from every *Streptomyces* strain were separately inoculated into MYG medium for mycelium growth. After cultivation for 72 h, total RNA was extracted using TRIzol (Thermo Fisher, Waltham, MA, United States). Reverse transcription was completed using a first-strand cDNA synthesis kit (Bio-Rad, Carlsbad, CA, United States). SYBR Green PCR Master Mix (Bio-Rad) was used for RT-qPCR. Primers were designed with an online tool.^1^ The reaction mixtures for RT-qPCR included 1 μL of cDNA templates, 1 μL of primers, 3 μL of ddH_2_O and 5 μL of SYBR Green PCR Master Mix and were assayed using the following program: 2 min at 50°C and 3 min at 95°C for one cycle; 15 s at 95°C, 30 s at 62°C and 30 s at 72°C for 30 cycles; and 10 min at 72°C for a final cycle. The endogenous *hrdB* gene was used as an internal control. The transcription levels of the other genes were normalized to the control.

### Fermentation and HPLC Analysis

After plasmid clearance, the culture of vegetative mycelium was spread over M-ISP4 plates and cultivated at 30°C for 4–7 days. The spores were then scraped and counted by spreading appropriate dilutions on plates and counting single colonies that had grown for 3 days. For daptomycin fermentation, 1 × 10^9^ *Streptomyces* spores were inoculated into 15 mL of MYG medium and grown at 30°C for 48 h as the seed culture. Then, the seed culture was inoculated in 500 mL of fermentation medium (40 g/L dextrin, 5 g/L casein, 80 g/L glucose, and 5 g/L MgSO_4_, pH 7.5) at 30°C for 9 days. After 48 h of fermentation, decanoate (final concentration 0.05%, w/v) was added every 12 h until the end of the fermentation period. After fermentation, the broth was centrifuged at 10,000 rpm for 30 min. The supernatant was then mixed with an equal volume of ethyl acetate to extract the metabolites. Next, the organic phase was dried under a vacuum. Finally, the metabolites were redissolved in 4 mL of methanol and filtered through a 0.22-μm membrane before the HPLC analysis.

The metabolites were analyzed on an HPLC system (Agilent Technologies Inc., Carpinteria, CA, United States) with an Agilent C18 reverse-phase column (internal diameter 4.6 mm × 250 mm, 5-μm particle size, Agilent Technologies, Inc.) at room temperature. The flow rate was 1 mL/min, and the absorbance of the eluate was monitored at 223 nm. The mobile phase, which was buffered with 0.01% formic acid, was initially maintained at a 75:25 water/acetonitrile for 5 min, followed by a linear gradient of 100% acetonitrile for 20 min.

### Statistical Analysis

The transformation frequency was calculated based on the ratio of the number of exconjugants to the number of spores used for conjugation. The experiments were performed in triplicate. The editing efficiency was calculated with the equation for editing efficiency (EF) = number of edited colonies evaluated by PCR/total number of evaluated colonies × 100%. All the colonies that showed double bands of WT and mutant products were calculated as wild type, and they were not included in the edited colonies. The editing efficiencies were calculated based on three replicates. Significant differences between the two groups were analyzed using *t* test. *p* < 0.05 was considered to indicate statistical significance.

## Results

### Design and Construction of Three CRISPR-*Fn*Cas12a Systems

**FIGURE 2:**
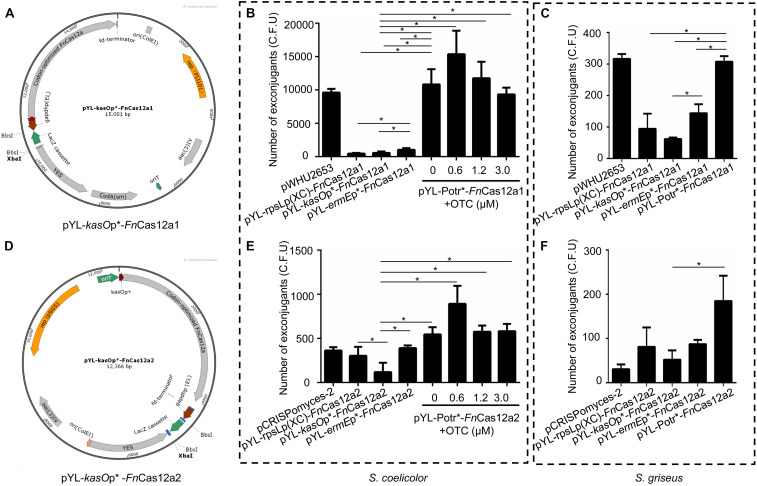
*Fn*Cas12a1 and *Fn*Cas12a2 systems and their transformation frequencies. **(A)** pYL-*kasO*p*-*Fn*Cas12a1 system. Notable features include the *kasO*p*-driven *Fn*Cas12a, the pIJ101 replicon, the *aac(3)IV* selection marker, the *codA(sm)* selection marker, a yeast helper fragment, the *Bbs*I-flanked *lacZ* cassette controlled by the gapdhp(EL) promoter and the B1006 terminator. **(B,C)** The numbers of exconjugants on plates after transfer of *Fn*Cas12a1 system plasmids to *S. coelicolor* and *S. griseus* for 7 days. **(D)** pYL-*kasO*p*-*Fn*Cas12a2 system. Most of the features of the pYL-*kasO*p*-*Fn*Cas12a2 system are the same as those of the pYL-*kasO*p*-*Fn*Cas12a1 system, excluding the temperature-sensitive pSG5 origin and the oop terminator of the *lacZ* cassette. The pYL-*kasO*p*-*Fn*Cas12a2 system lacks a *traJ* gene. **(E–F)** The numbers of exconjugants on plates after transfer of *Fn*Cas12a2 system plasmids to *S. coelicolor* and *S. griseus* for 7 days. The experiments were performed in triplicate. * Indicates a significant difference.

In previous studies, the pCRISPomyces-2 system (Cobb et al., 2015) and the pWHU2653 system (Zeng et al., 2015) have exhibited high editing efficiencies in model *Streptomyces* strains. To develop efficient and versatile genome editing tools in multiple *Streptomyces* strains, two *Fn*Cas12a systems were constructed: one based on the pWHU2653 system (Zeng et al., 2015) (CRISPR*-Fn*Cas12a1) and the other based on the pCRISPomyces-2 system (Cobb et al., 2015) (CRISPR*-Fn*Cas12a2). The *Fn*Cas12a1 system carries the selection markers *codA(sm)* (Dubeau et al., 2009) and *aac(3)IV*, the *rep*(pIJ101) replicon, and the B1006 terminator that terminates the transcription of the crRNA cassette, while the *Fn*Cas12a2 system carries only the selection marker *aac(3)IV*, the *pSG5* origin of replication, and the fd terminator that terminates the transcription of the crRNA cassette (Supplementary Table 2). Three constitutive promoters, *kasO*p^∗^ (Wang et al., 2013), rpsLp(XC) (Shao et al., 2013), and *ermE*p^∗^ (Luo et al., 2015a), and an inducible Potr^∗^ system were selected to control the expression of *Fn*Cas12a in each system. Overall, 8 plasmids were constructed: pYL-*kasO*p^∗^-*Fn*Cas12a1, pYL-rpsLp(XC)-*Fn*Cas12a1, pYL-*ermE*p^∗^-*Fn*Cas12a1, pYL-Potr^∗^-*Fn*Cas12a1, pYL-*kasO*p^∗^-*Fn*Cas12a2, pYL-rpsLp(XC)-*Fn*Cas12a2, pYL-*ermE*p^∗^-*Fn*Cas12a2, and pYL-Potr^∗^-*Fn*Cas12a2 (Figures 2A,D, Supplementary Figure 1, and Table 3). The CRISPR-*F*nCas12a3 system was constructed based on the *Fn*Cas12a2 system by replacing the WT *Fn*Cas12a protein with the *Fn*Cas12a variant EP16.

**TABLE 1:** Deletion efficiencies in the *actII*-*orf4* gene deletion experiments by using two *Fn*Cas12a systems.

Plasmid	Editing efficiency
pYL-*kasO*p^∗^-*Fn*Cas12a1-*actII*-*orf4*-DR19	28.6% ± 11.7%
pYL-*kasO*p^∗^-*Fn*Cas12a1-*actII*-*orf4*-DR36	76.2% ± 6.7%
pYL-rpsLp(XC)-*Fn*Cas12a1-*actII*-*orf4*-DR19	33.3% ± 6.7%
pYL-rpsLp(XC)-*Fn*Cas12a1-*actII*-*orf4*-DR36	71.4% ± 11.7%
pYL-*ermE*p^∗^-*Fn*Cas12a1-*actII*-*orf4*-DR19	14.3% ± 11.7%
pYL-*ermE*p^∗^-*Fn*Cas12a1-*actII*-*orf4*-DR36	23.8% ± 6.7%
pYL-Potr^∗^-*Fn*Cas12a1-*actII*-*orf4*-DR19	9.5% ± 6.7%
pYL-Potr^∗^-*Fn*Cas12a1-*actII*-*orf4*-DR36	28.6% ± 14.3%
pYL-*kasO*p^∗^-*Fn*Cas12a2-*actII*-*orf4*-DR19	80.2% ± 6.3%
pYL-*kasO*p^∗^-*Fn*Cas12a2-*actII*-*orf4*-DR36	100.0% ± 0.0%
pYL-rpsLp(XC)-*Fn*Cas12a2-*actII*-*orf4*-DR19	81.0% ± 17.8%
pYL-rpsLp(XC)-*Fn*Cas12a2-*actII*-*orf4*-DR36	94.4% ± 7.9%
pYL-*ermE*p^∗^-*Fn*Cas12a2-*actII*-*orf4*-DR19	14.3% ± 0.0%
pYL-*ermE*p^∗^-*Fn*Cas12a2-*actII*-*orf4*-DR36	28.6% ± 11.7%
pYL-Potr^∗^-*Fn*Cas12a2-*actII*-*orf4*-DR19	23.8% ± 6.7%
pYL-Potr^∗^-*Fn*Cas12a2-*actII*-*orf4*-DR36	21.4% ± 7.1%

All the above plasmids were transformed into different *Streptomyces* strains. Over 10,000 exconjugants were generated by the transformation of *S. coelicolor* with pYL-Potr^∗^-*Fn*Cas12a1 with or without addition of oxytetracycline (OTC), a value that was significantly greater than the number of exconjugants generated by plasmids pYL-*kasO*p^∗^-*Fn*Cas12a1, pYL-rpsLp(XC)-*Fn*Cas12a1, and pYL-*ermE*p^∗^-*Fn*Cas12a1 (Figure 2B). Meanwhile, 0.6 μM OTC in M-ISP4 medium induced the greatest number of exconjugants (12453 ± 4582). Higher OTC concentrations resulted in fewer exconjugants. Similarly, introducing pYL-Potr^∗^-*Fn*Cas12a1 into *S. griseus* generated 308 ± 13 exconjugants. Other *Fn*Cas12a1 plasmids were also transformed into *S. griseus* and generated 95 ± 39 (pYL-rpsLp(XC)-*Fn*Cas12a1), 62 ± 4 (pYL-*kasO*p^∗^-*Fn*Cas12a1), and 143 ± 23 (pYL-*ermE*p^∗^-*Fn*Cas12a1) exconjugants, respectively (Figure 2C).

A small number of *S. coelicolor* exconjugants (178 ± 27) were generated after introducing pYL-*kasO*p^∗^-*Fn*Cas12a2, while more exconjugants (303 ± 83 and 391 ± 21, respectively) were generated after introducing pYL-rpsLp(XC)-*Fn*Cas12a2, pYL-*ermE*p^∗^-*Fn*Cas12a2, and pYL-Potr^∗^-*Fn*Cas12a2. The transformation of the plasmid pYL-Potr^∗^-*Fn*Cas12a2 in the absence of OTC resulted in more exconjugants (546 ± 66) than the transformation of other *Fn*Cas12a2 system plasmids with constitutive promoters. Moreover, in the presence of OTC at a final concentration of 0.6 μM, the transformation of pYL-Potr^∗^-*Fn*Cas12a2 generated the greatest number of exconjugants (892 ± 166) (Figure 2E). Similarly, the introduction of pYL-Potr^∗^-*Fn*Cas12a2 into *S. griseus* generated the most exconjugants in the absence of OTC (Figure 2F). A comparison of editing efficiencies between two systems showed that all *Fn*Cas12a1 system plasmids exhibited significantly higher transformation frequencies than *Fn*Cas12a2 system plasmids. The transformation frequencies of the *Fn*Cas12a1 system plasmids were higher than those of the *Fn*Cas12a2 system plasmids in *S. griseus*. However, there were no statistically significant differences in *S. griseus* unlike *S. coelicolor* (Supplementary Figure 2).

Additionally, *Fn*Cas12a systems controlled by the promoters *ermE*p^∗^ and Potr^∗^ showed significantly higher transformation frequencies than those controlled by *kasO*p^∗^ (Figures 2B,C,E,F). These results are consistent with those of a study by Ungerer and Pakrasi, which revealed that a vector carrying *Fn*Cas12a yielded fewer colonies than an empty vector. *Fn*Cas12a is toxic to the host, and host toxicity increases with increasing *Fn*Cas12a promoter strength. Therefore, we chose the *Fn*Cas12a1 system driven by the Potr^∗^ or *ermE*p^∗^ promoter to perform genome editing in *Streptomyces* strains that are difficult to be transformed.

### Optimization of the CRISPR-*Fn*Cas12a Systems

To optimize the CRISPR-*Fn*Cas12a systems, we assessed the impacts of the DR length and the *Fn*Cas12a transcription level on genome editing efficiency. In some hosts, a shorter DR length resulted in a higher editing efficiency, as observed for *Fn*Cas12a in *Saccharomyces cerevisiae* (Swiat et al., 2017) and for *As*Cas12a in mammalian cells (Zetsche et al., 2017). In some other hosts, such as *Synechococcus* UTEX 2973 (Ungerer and Pakrasi, 2016), *Streptomyces* sp. NRRL S−244 (Yeo et al., 2019), and *Streptomyces albus* J1074 (Yeo et al., 2019), 36-nt DRs have shown high editing efficiency. However, there have been no reports focusing on the impact of the DR length on the editing efficiency in *Streptomyces* strains.

**FIGURE 3:**
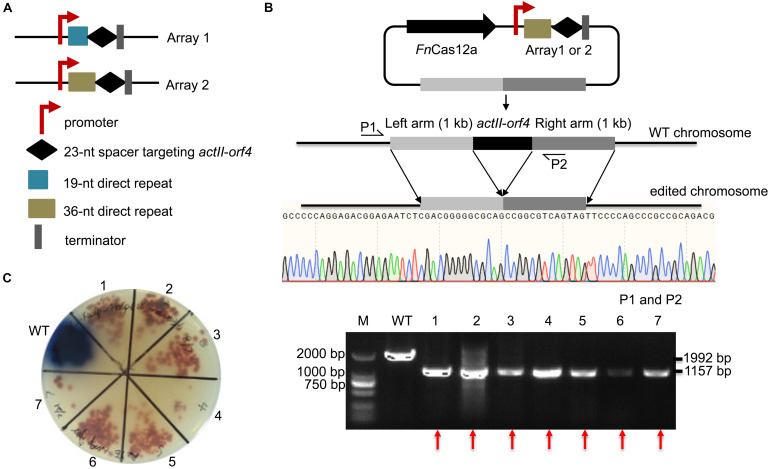
Evaluation of the single-gene deletion efficiency. **(A)** Two arrays were designed for deletion of the *actII-orf4* gene. Array 1 contained the gapdhp(EL) promoter, a 19-nt DR sequence, a 23-nt spacer and the oop terminator. Array 2 contained similar elements, except that it contained a 36-nt DR. **(B)** Schematic of gene editing using *Fn*Cas12a with a single DR-spacer cassette and a single editing template. The editing template repaired the DSB cut induced by *Fn*Cas12a via HDR. Sequencing of PCR products amplified from the edited strains indicated the complete deletion of the *actII-orf4* gene with no undesired mutations. P1 and P2 indicate the locations of the primers used to validate the deletion. **(C)** Phenotypic evaluation and PCR evaluation of the WT strain *S. coelicolor* A3(2) and 7 randomly selected exconjugants. Red pigmentation was produced by the *actII-orf4-*deletion strains, but not by WT strains. The 1,992- and 1,157-bp replicons were amplified from the WT and *actII-orf4-*deletion strains, respectively. The experiments were performed in triplicate. Red arrows indicate the successfully deleted colonies.

To confirm the impact of DR length on *Fn*Cas12a-mediated genome editing efficiency in *Streptomyces* strains, we chose two crRNA arrays with different DR lengths to guide *actII-orf4* gene editing in *S. coelicolor*. Array 1 contained a 19-nt DR and a 23-nt spacer, while array 2 contained a 36-nt DR and a 23-nt spacer (Figure 3A). CRISPR array 1 or 2 and an editing template were introduced into every pYL-*Fn*Cas12a plasmid to obtain a series of *actII-orf4* deletion plasmids (Figure 3B and Supplementary Table 3). When controlled by *kasO*p^∗^, *Fn*Cas12a pairing with array 2 showed a significantly higher deletion efficiency than that pairing with array 1 for both *Fn*Cas12a1 and *Fn*Cas12a2 systems in *S. coelicolor* (Table 1 and Supplementary Figures 3A,B). Specifically, plasmid pYL-*kasO*p^∗^-*Fn*Cas12a2-*actII-orf4*-DR36 led to a loss of blue pigment and complete *actII-orf4* deletion with the highest editing efficiency of 100% (Figure 3C and Table 1). In addition, when driven by *kasO*p^∗^ or rpsLp(XC), the *Fn*Cas12a2 system showed significantly higher editing efficiencies than *Fn*Cas12a1 system for both 19-nt and 36-nt DRs (Supplementary Figure 4). In summary, pYL-*kasO*p^∗^-*Fn*Cas12a2 carrying the 36-nt DR showed the highest and most stable editing efficiency among all *Fn*Cas12a plasmid combinations (Table 1 and Supplementary Figure 4). Therefore, pYL-*kasO*p^∗^-*Fn*Cas12a2 with a 36-nt DR was deemed the most suitable system for genome editing.

To explore the effects of *Fn*Cas12a expression level on genome editing efficiency, we measured the transcription levels of *Fn*Cas12a in *S. coelicolor*. We found that the transcription levels of *Fn*Cas12a were significantly higher under the control of *kasO*p^∗^ than under the control of other promoters or the inducible system for both *Fn*Cas12a1 and *Fn*Cas12a2 systems (Supplementary Figure 5). With regard to editing efficiency, the plasmids carrying the 36-nt crRNA and *Fn*Cas12a driven by the strong promoter *kasO*p^∗^ resulted in higher editing efficiencies than those driven by a weak constitutive promoter (*ermE*p^∗^) or an inducible system (Potr^∗^) (Table 1, Supplementary Figure 6, and Tables 3, 4). Notably, the inducible system has been reported to be fully induced by OTC at a final concentration of 3 μM in *S. coelicolor* M1146 (Wang et al., 2016). Thus, we performed transformations on M-ISP4 plates supplemented with OTC at final concentrations of 0, 0.6, 1.2, and 3.0 μM to maintain the Potr^∗^ system at low, medium and high activity levels. Among the four OTC concentrations, 3.0 or 1.2 μM OTC was sufficient to induce a high editing efficiency of pYL-Potr^∗^-*Fn*Cas12a1-*actII-orf-*DR19 (23.8% ± 13.5%) or pYL-Potr^∗^-*Fn*Cas12a1-*actII-orf-*DR36 (28.6% ± 11.7%). In the *Fn*Cas12a2 system, 1.2 μM OTC supplementation was sufficient to induce a high editing efficiency of pYL-Potr^∗^-*Fn*Cas12a2-*actII-orf4*-DR19 (35.7% ± 7.1%) or pYL-Potr^∗^-*Fn*Cas12a2-*actII-orf4*-DR36 (69.1% ± 2.4%) (Supplementary Table 4). Then, we measured the transcription levels of *Fn*Cas12a driven by the inducible Potr^∗^ system in the presence of 0, 0.6, 1.2 and 3.0 μM OTC. Significantly higher transcription levels of the *Fn*Cas12a were produced by both the *Fn*Cas12a1 and *Fn*Cas12a2 systems in the presence of 0.6, 1.2 and 3.0 μM OTC than in the absence of OTC (Supplementary Figures 5A,B). Pearson’s correlation coefficients were calculated to explore the correlations between *Fn*Cas12a transcription levels and editing efficiencies. The analysis revealed that *Fn*Cas12a expression and the editing efficiency were positively correlated with the *Fn*Cas12a1 (correlation coefficient: *p* = 0.050, *r* = 0.750) and *Fn*Cas12a2 systems (correlation coefficient: *p* = 0.036, r = 0.790) when pairing with the 36-nt DR-containing crRNA (Supplementary Figures 5C,D). Briefly, elevated *Fn*Cas12a expression leads to increased genome editing efficiency of *Fn*Cas12a pairing with the 36-nt DR-containing crRNA.

### The Ability of *Fn*Cas12a to Delete Large Chromosomal Fragments

**FIGURE 4:**
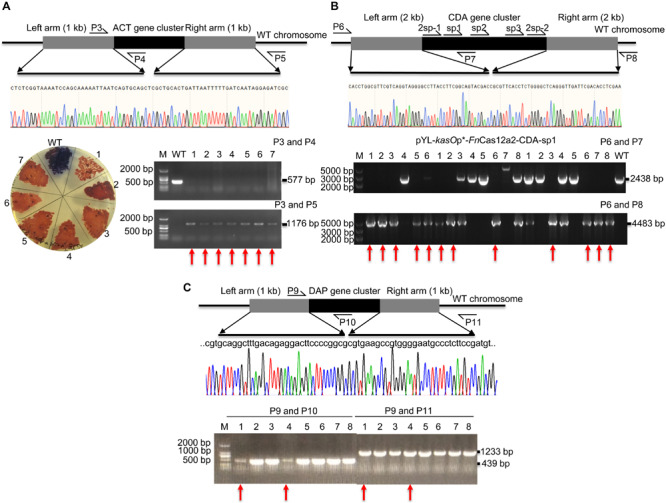
Evaluation of a large-fragment deletion via the *Fn*Cas12a2 system. **(A)** Identification of the ACT gene cluster deletion. Red pigment was produced by the ACT-deletion strains but not by the WT strains. The primers P3 and P4 produced a 577-bp amplicon for the WT strains and no products for the ACT-deletion strains. The primers P3 and P5 generated an 1,176-bp amplicon for the ACT-deletion strains and no products for the WT strains. The sequencing data for the 1,176-bp amplicon showed the complete deletion of the ACT gene cluster. **(B)** Evaluation of CDA gene cluster deletion. For single cuts, three single spacers, sp1, sp2, and sp3, were selected. For double cuts, the paired spacers 2sp-1 and 2sp-2 were selected. The primers P6 and P7 produced a 2,438-bp amplicon for the WT strains and no products for the CDA-deletion strains. The primers P6 and P8 generated a 4,483-bp amplicon for the CDA-deletion strains, but no products for the WT strains. **(C)** Identification of DAP gene cluster deletion. The primers P9 and P10 generated a 439-bp amplicon for the WT strains, but no products for the DAP-deletion strains. The primers P9 and P11 generated a 1,233-bp amplicon for the DAP-deletion strains but no products for the WT strains. The sequencing data for the 1,233-bp amplicon showed the complete deletion of the DAP gene cluster. The experiments were performed in triplicate. Red arrows indicate the successfully deleted colonies.

pYL-*kasO*p^∗^-*Fn*Cas12a2 carrying a 36-nt DR has been proven to be efficient for genome editing in *Streptomyces* strains. Thus, the ability of *Fn*Cas12a to delete large DNA fragments was evaluated. The actinorhodin biosynthesis gene cluster (ACT, 21.4 kb) and Ca^2+^-dependent antibiotic biosynthesis gene cluster (CDA, 82.8 kb) in *S. coelicolor* and the daptomycin biosynthesis gene cluster (DAP, 127.6 kb) in *S. roseosporus* were selected. For single cuts, one to three spacers were selected for each cluster. The ACT-deletion strains produced red pigment on R2YE plates, while the WT strains produced blue pigment (Figure 4A). Subsequently, the PCR results showed that the 577-bp bands were amplified from the genomic DNA of WT strains but not from that of the edited strains. In addition, the 1,176-bp bands were amplified from the genomic DNA of successfully edited strains but were not amplified from the genomic DNA of WT strains (Figure 4A). The sequencing of the 1,176-bp fragments indicated that the ACT gene cluster was completely deleted from the genome of *S. coelicolor*. The deletion efficiency of the 21.4-kb gene fragment was 92.9% ± 7.2% (Supplementary Table 5). The efficiencies decreased with increasing deletion fragment sizes. The deletion efficiencies of the CDA gene cluster were 55.6% ± 7.9% (sp1), 18.8% ± 6.3% (sp2), and 25.0% ± 0% (sp3) (Supplementary Table 5 and Figure 4B). The deletion efficiencies of the DAP gene cluster were 25.0% ± 0% (sp1) and 0% (sp2) (Supplementary Table 5 and Figure 4C). To increase editing efficiency, we also attempted to introduce double cuts in the CDA gene cluster using an *Fn*Cas12a2 system carrying two crRNA cassettes (36-nt DR+2sp-1+36-nt DR+2sp-2). Two spacers (2sp-1 and 2sp-2) flanking the edges of the CDA gene cluster were selected to cut the two loci in the cluster. Two 2-kb arms homologous to the corresponding upstream and downstream sequences of the target gene clusters were introduced into the plasmids to repair the double-strand breaks at the edges of the CDA gene cluster. However, no increase in the editing efficiency was observed (25.0% ± 0%) (2sp) (Supplementary Table 5 and Figure 4B).

### Application of the CRISPR-*Fn*Cas12a System for Multiplex Genome Editing in Streptomyces Species

**FIGURE 5:**
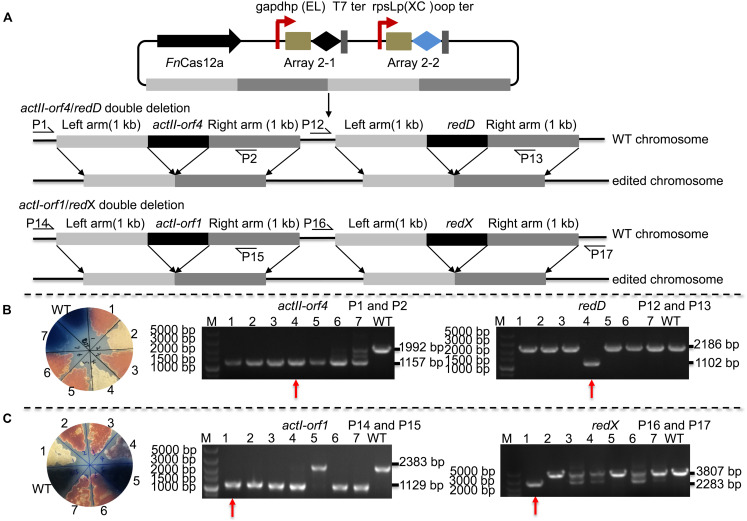
Strategy and evaluation of double deletion via the *Fn*Cas12a2 system. **(A)** Strategy used for double deletion. Each plasmid for *actII-orf4*/*redD* double deletion or *actI-orf1*/*red*X double deletion carried two arrays and two editing templates. Array 2-1, including a 36-nt DR and spacer 1, was controlled by gapdhp(EL) and terminated by the T7 terminator; array 2-2, including a 36-nt DR and spacer 2, was controlled by rpsLp(XC) and terminated by the oop terminator. The template for every target consisted of left and right arms corresponding to upstream and downstream sequences of the target gene, respectively. P1, P2, P12, P13, P14, P15, P16, and P17 indicate the locations of the primers used to assess the deletions. **(B)** Seven randomly selected exconjugants carrying the *actII-orf4*/*redD* double-deletion plasmid were selected for verification by phenotype screening and PCR. The double-deletion mutant strains produced no pigment on R2YE. For the *actII-orf4*/*redD* double-deletion mutant strains, the primers P1 and P2 produced an 1,157-bp amplicon, while the primers P12 and P13 produced an 1,102-bp amplicon; in contrast, for the strains in which double deletion failed, the primers produced a 2,188-bp amplicon or a 1,992-bp amplicon. **(C)** Similarly, seven randomly selected exconjugants carrying the *actII-orf1*/*redX* double-deletion plasmid were selected for verification. For the *actI-orf1*/*redX* double-deletion mutant strains, the primers P14 and P15 produced an 1,129-bp amplicon, while the primers P16 and P17 produced a 2,283-bp amplicon; for the strains in which double deletion failed, the primers produced a 2,383-bp amplicon and a 3,807-bp amplicon. The double-deletion mutants are indicated by red arrows. The experiments were performed in triplicate.

Editing multiple genes step by step in *Streptomyces* is labor-intensive and time-consuming since the *Streptomyces* growth cycle is relatively long. Thus, an efficient tool is needed to perform multigene editing. pYL-*kasO*p^∗^-*Fn*Cas12a2 was used to assess the possibility of CRISPR/*Fn*Cas12a-mediated multiplex gene deletion. We constructed an *actII*-*orf4*/*redD* double-deletion mutant and an *actI*-*orf1*/*redX* double-deletion mutant based on the pYL-*kasO*p^∗^-*Fn*Cas12a2 system. Both double-deletion constructs contained two arrays. Array 2-1 consisted of the gapdhp(EL) promoter, DR-spacer1 and the T7 terminator. Array 2-2 consisted of the rpsLp(XC) promoter, DR-spacer2 and oop terminator (Figure 5A). *ActII*-*orf4* and *redD* are pathway-specific regulatory genes, and *actI*-*orf1* and *redX* are beta-ketoacyl synthase genes. *ActII*-*orf4* and *actII*-*orf1* are responsible for actinorhodin biosynthesis, while *redD* and *redX* contribute to undecylprodigiosin biosynthesis in *S. coelicolor*. *S. coelicolor* lacking *actII*-*orf4* or *actI*-*orf1* failed to synthesize actinorhodin and produced a red pigment on R2YE medium. *RedD* or *redX* deletion abolished undecylprodigiosin synthesis and resulted in a blue color on R2YE medium (Figures 5B,C). The success of double deletion of *actII*-*orf4* and *redD* was evaluated using PCR. For the double deletions of *actII*-*orf4* and *redD*, the generation of an 1,157-bp band indicated the deletion of *actII*-*orf4*, and the amplification of an 1,102-bp band indicated the deletion of *redD*. Among the seven randomly selected exconjugants, exconjugant 4 harbored the correct double deletion (Figure 5B). For the double deletion of *actI*-*orf1* and *redX*, the amplification of an 1,129-bp band indicated the deletion of *actI*-*orf1*, and the amplification of a 2,283-bp band indicated the deletion of *redX*. Among the seven randomly selected exconjugants, exconjugant 1 harbored the correct double deletion, and colonies 3, 4, 6, and 7 showed mixtures of the *redX*-deletion mutant and the WT strain (Figure 5C). This phenomenon is common in microbes and has been called “incomplete genome editing” (Huang et al., 2015b; Ungerer and Pakrasi, 2016). The colonies that showed double bands with WT and mutant sizes were not classified as mutant colonies. The double-deletion efficiencies of *actII*-*orf4*/*redD* and *actI*-*orf1*/*redX* were 14.3% ± 0% and 14.3% ± 0%, respectively (Supplementary Table 5 and Supplementary Figure 7).

### Precise Genome Editing With the CRISPR-*Fn*Cas12a Systems

pYL-*ermE*p^∗^-*Fn*Cas12a1 and pYL-Potr^∗^-*Fn*Cas12a1 have been indicated to have increased transformation frequencies in *Streptomyces*. Thus, the genome editing efficiencies of these constructs were tested in *S. hygroscopicus* NRRL5491, in which it is difficult to perform genetic editing. Rapamycin is a macrolide immunosuppressant produced by *S. hygroscopicus* NRRL5491 and *Actinoplanes* sp. N902-109 (Huang et al., 2015a), and it has been approved as a treatment for select conditions by the FDA (Arriola Apelo and Lamming, 2016). In addition, rapamycin exhibits antifungal and anticancer activities. According to a previous study, *Actinoplanes* sp. N902-109 carries an additional *rapTH* gene and exhibits higher production of rapamycin than *S. hygroscopicus* NRRL5491. *RapTH* encodes a homolog of type II thioesterase (Huang et al., 2015a). The *rapTH* gene has been proposed to play an important role in rapamycin generation. To introduce *rapTH* into *S. hygroscopicus* NRRL5491, we inserted the rpsLp(CF) promoter in front of *rapTH* and inserted the *ermE*p^∗^ promoter in front of *rapQ* in constructs based on pYL-*ermE*p^∗^-*Fn*Cas12a1 and pYL-Potr^∗^-*Fn*Cas12a1 (Supplementary Figure 8A). The insertion fragment contained the *ermE*p^∗^ promoter (282 bp), the *rapTH* gene (756 bp) and the rpsLp(CF) promoter (302 bp) (a total of 1,340 bp) (Supplementary Figure 8A). Eight to twelve single colonies were selected and evaluated. A 2,277-bp band was amplified from the genomic DNA of WT strains, while a 3,668-bp band was amplified from genomic DNA of successfully edited strains (Supplementary Figure 8B). The maximum insertion efficiency of the two promoters and the *rapTH* gene was 25.0% when pYL-Potr^∗^-*Fn*Cas12a1 was used in the presence of 1.2 μM OTC (Supplementary Table 5).

**FIGURE 6:**
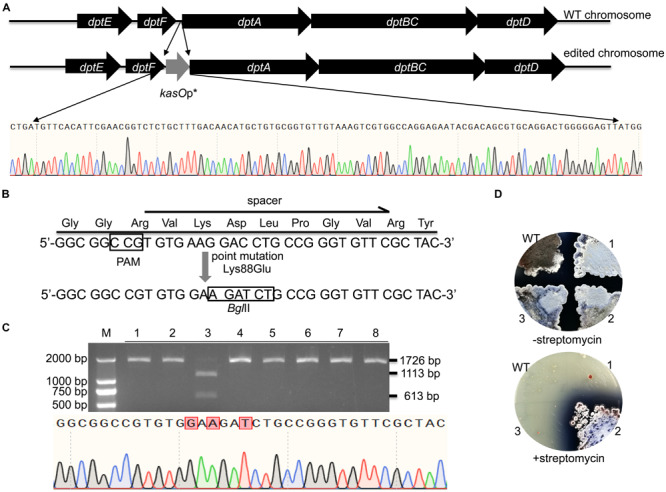
Evaluation of CRISPR-*Fn*Cas12a3-mediated precise genome editing. **(A)** Strategy and evaluation of the *kasO*p* insertion by CRISPR-*Fn*Cas12a3. The sequencing results showed the successful insertion of the promoter *kasO*p*. **(B)** Evaluation of the precise mutations caused by CRISPR-*Fn*Cas12a3 with the CCG PAM. The Lys88Glu mutation and GAC-to-GAT mutations were introduced into the spacer region in the DSB repair template. **(C)** The 1,726-bp PCR products amplified from the mutants were digested into 1,113-bp and 613-bp fragments by *Bgl*II, while the WT amplicons were not digested. The sequencing data for the PCR product of the *rpsL* gene revealed the three mutations that are highlighted in red. **(D)** Three randomly selected exconjugants and one WT *S. coelicolor* strain were picked and streaked onto R2YE plates with or without 100 μg/mL streptomycin. Three days later, the WT strains were sensitive to 100 μg/mL streptomycin, while the mutant strains were resistant to 100 μg/mL streptomycin. The experiments were performed in triplicate.

WT *Fn*Cas12a requires a TTN PAM sequence, which may limit its application in *Streptomyces* strains with high-GC-content genomes. For gene deletion, a TTN or NGG PAM can easily be selected in the whole gene reading frame. Thus, there are no significant restrictions in the use of CRISPR-Cas9 or CRISPR-Cas12a. However, the selection region is sometimes limited for insertions or site mutations. Therefore, the use of an NGG or TTN PAM may be restricted. Daptomycin, a cyclic lipopeptide produced in *S. roseosporus*, shows significant activity against Gram-positive pathogens, such as methicillin-resistant *Staphylococcus aureus* (MRSA) (Vilhena and Bettencourt, 2012). The daptomycin biosynthetic pathway contains three nonribosomal peptide synthetase (NRPS) genes, *dptA*, *dptBC* and *dptD* (Miao et al., 2005). To increase the production of daptomycin in *S. roseosporus*, we planned to introduce *kasO*p^∗^ in front of the *dptA* gene, which encodes the first subunit of NRPS (Figure 6A). However, no TTN PAM sequences suitable for *Fn*Cas12a recognition were located in front of the NRPS genes of daptomycin. Therefore, it was impossible for us to introduce a strong promoter in front of the NRPS genes via the *Fn*Cas12a system with WT *Fn*Cas12a. To overcome this limitation, we applied a CRISPR-Cas12a3 system containing the *Fn*Cas12a mutant EP16, which was previously generated by our laboratory (Wang et al., 2019). Among the tested variants, the *Fn*Cas12a mutant EP16 (N607R/K613V/N617R/K180S/K660R/D616N) exhibited the best recognition capabilities *in vitro*, as it could recognize YN (Y = C or T), TAC and CAA PAMs. We selected three spacers adjacent to CCG, CCA and ATC PAMs with which the *Fn*Cas12a variant EP16, but not WT *Fn*Cas12a, worked well *in vitro*. The *kasO*p^∗^ promoter insertion efficiencies of CRISPR-*Fn*Cas12a3 with CCG, CCA and ATC PAMs were 50.0% ± 12.5%, 40.0% ± 17.4%, and 23.6% ± 8.6%, respectively (Supplementary Table 5). In summary, the CRISPR-*Fn*Cas12a3 system provides opportunities to select suitable PAMs for precise genome editing in GC-rich organisms.

After we introduced *kasO*p^∗^ in front of the daptomycin biosynthesis genes, we performed plasmid clearance to generate the strain *S. roseosporus*/PkasA (Figure 9A). Introduction of *kasO*p^∗^ increased the transcription levels of the *dptA*, *dptBC* and *dptD* genes by 1. 83-, 1.12- and 2.04-fold, respectively (Supplementary Figure 9A). Compared with WT *S. roseosporus*, *S. roseosporus*/PkasA showed increased production of daptomycin (Supplementary Figures 9B,C).

In addition to insertions, CRISPR-*Fn*Cas12a3 was also applied to generate precise site mutations. *RpsL*(SCO4659) encodes the ribosomal protein S12 in *S. coelicolor* A3(2), and its specific site mutation (K88E) confers resistance to high concentrations of streptomycin (100 μg/mL) (Shima et al., 1996). Here, we introduced a GAA mutation (K88E) to ensure the resistance of *S. coelicolor* A3(2) to streptomycin (Figure 6B). Moreover, a specific C-to-T mutation was introduced at the 267th nucleotide of *rpsL* to introduce a *Bgl*II restriction site for rapid identification of the correct exconjugants (Figure 6B). To avoid undesirable re-editing, the double-strand break (DSB) was repaired using a template containing altered nucleotides at the 5′ end of the spacer sequence (Figure 6B). PCR amplicons of 1,726 bp were amplified from the genomes of eight exconjugants and digested separately with the *Bgl*II restriction enzyme. One 1,726-bp PCR amplicon was successfully digested into 1,113-bp and 613-bp fragments. Subsequent sequencing of the 1,726-bp PCR amplicon from exconjugant 3 indicated that the site mutations were successfully created (Figure 6C). The restriction digestion and DNA sequencing results showed a site mutation efficiency 12.5% ± 0% (Figure 6C and Supplementary Table 5). However, phenotype screening indicated that 33.3% of the *S. coelicolor* A3(2) colonies harboring pYL-*kasOp*^∗^-*Fn*Cas12a3-rpsL were resistant to streptomycin (Figure 6D). These results suggest that some strains failed to be mutated.

## Discussion

In the present study, we developed three useful genome editing tools with different advantages and applied them to examine their transformation frequencies and genome editing efficiencies in several *Streptomyces* species. The successful application of the CRISPR-*Fn*Cas12a3 system with expanded PAM recognition ability for precise insertions and site mutations overcomes the restricted applications of the TTN PAM in organisms with high GC contents. Its flexibility in PAM selection will promote the application of *Fn*Cas12a in *Streptomyces* species. The *Fn*Cas12a1 system worked well in *S. hygroscopicus*, in which CRISPR-Cas9 is ineffective. The *Fn*Cas12a2 system efficiently deleted large chromosomal fragments (∼128 kb) and was useful for deleting multiple genes. Altogether, these three systems have different advantages and are complementary to each other.

Due to the high GC content of the *Streptomyces* genome, it will not be easy for us to select a TTN PAM with high prediction scores. In particular, the number of suitable sequence region that can be targeted for insertions and site mutations is very limited. Thus, it is inconvenient to select a TTN PAM for large-scale genome engineering in *Streptomyces*. The *Fn*Cas12a mutant EP16 has a wide range of PAM recognition sites (60/64 sites), including YN (Y = C or T), TAC and CAA sites. EP16 cleaves target DNA by the PAMs CCG, CCA and ATC *in vitro* with high efficiencies of 97, 94 and 96%, respectively. However, the efficiencies of CRISPR-*Fn*Cas12a3 harboring EP16 with CCG, CCA and ATC recognition sites were only 50.0% ± 12.5%, 40.0% ± 17.4%, and 23.6% ± 8.6%, respectively, *in vivo*. This phenomenon is common. As shown in the studies by Zetsche et al. and Tu et al., *Fn*Cas12a recognizes TTN sites *in vitro* but frequently fails to recognize TTN sites in human cells (Zetsche et al., 2015; Tu et al., 2017). *Fn*Cas12a has been reported to prefer KYTV in human cells (Sun et al., 2018), but prefer TTTV PAMs in rice (Zhong et al., 2018). Moreover, our unpublished data from human HEK293T cells also show a similar phenomenon. This phenomenon may be attributable to the complex microenvironments in living organisms. For instance, post-translational modifications, such as acetylation (Ishigaki et al., 2017) and methylation (Huang et al., 2018), are common in *Streptomyces*. *Moraxella bovoculi* (Mb) Cas12a has been demonstrated to lose its cleavage functions upon the acetylation of the critical PAM recognition residue Lys635 (Dong et al., 2019) *in vitro*. Taken together, results indicate that the *Fn*Cas12a3 system overcomes the restricted applications of WT *Fn*Cas12a, which requires a TTN PAM for editing in *Streptomyces*. Although the editing efficiencies of the *Fn*Cas12a3 system were low, the identification of a new Cas12a variant (EP16) that works on a broad range of PAMs in *Streptomyces* is a very important step forward. This powerful tool will enable researchers to generate desired insertions and precise site mutations and will also enable the activation of biosynthetic pathways to generate valuable natural products.

In a previous study, conservation was discovered at the 3′ end of the DR sequence among all *Fn*Cas12a family proteins (Zetsche et al., 2015). In addition, 19-nt DR-containing crRNA cassettes have been proven to exhibit good editing efficiencies *in vitro* (Wang et al., 2019) and in some host cells, such as human HEK293T cells (Zetsche et al., 2017) and *S. cerevisiae* (Swiat et al., 2017). In another study, a 36-nt DR was applied for markerless editing in *Cyanobacteria* species (Ungerer and Pakrasi, 2016). Although *Fn*Cas12a has been used in many hosts, to the best of our knowledge, only one report has focused on the impacts of the DR length on the editing efficiency in *Saccharomyces cerevisiae* (Swiat et al., 2017). In the current study, a crRNA containing a 19-nt DR led to much higher editing efficiency than that containing a 36-nt DR in *Saccharomyces cerevisiae*. In *Streptomyces* species, when *Fn*Cas12a was controlled by strong constitutive promoters, the crRNA containing a 36-nt DR led to high editing efficiency. However, when *Fn*Cas12a was controlled by a weak promoter or an inducible system, significant differences in the editing efficiencies resulting from 36-nt DR-containing crRNA and 19-nt DR-containing crRNA were not observed (Table 1 and Supplementary Figure 3). Therefore, the impact of the DR length is different for different plasmid systems or hosts. Thus, the impact of the DR length should be evaluated whenever Cas12a-mediated genome editing is performed for the first time in a specific organism.

The transformation frequencies and editing efficiencies of the *Fn*Cas12a1 and *Fn*Cas12a2 systems were significantly different. The plasmids constructed from the *Fn*Cas12a1 system induced markedly higher transformation frequencies than those constructed from the *Fn*Cas12a2 system. A comparison of the elements related to transformation revealed that the *Fn*Cas12a1 system carries a *traJ* gene. This gene encodes an activator of the transfer (*tra*) operon, which is crucial for the transfer region of the fertility factor (Will and Frost, 2006). After the *traJ* gene was deleted from the *Fn*Cas12a1 system, the transformation frequency was significantly decreased (Supplementary Figure 10). In this case, the *traJ* gene in *Fn*Cas12a1 system was required for its high transformation frequency. Moreover, the *Fn*Cas12a1 system contains the counterselection marker *codA(sm)*, which saves a substantial amount of time during plasmid elimination (Zeng et al., 2015). Thus, the *Fn*Cas12a1 system is more suitable than the *Fn*Cas12a2 system for applications in some strains with low transformation frequencies. On the other hand, Wei et al. (2017) found that strong terminators are responsible for high gene expression. To explore whether the different terminators of crRNA arrays contribute to the different editing efficiencies between *Fn*Cas12a1 system and *Fn*Cas12a2 system, we exchanged the terminators of crRNA arrays of the two systems to construct the plasmids pYL-*kasO*p^∗^-*Fn*Cas12a1-*actII-orf4*-DR36-oop and pYL-*kasO*p^∗^-*Fn*Cas12a2-*actII-orf4*-DR36-B1006. The pYL-*kasO*p^∗^-*Fn*Cas12a1-*actII-orf4*-DR36-oop plasmid led to significantly higher editing efficiency (95.2% ± 6.7%) than the pYL-*kasO*p^∗^-*Fn*Cas12a1-*actII-orf4*-DR36 plasmid (76.2% ± 6.7%), and pYL-*kasO*p^∗^-*Fn*Cas12a2-*actII-orf4*-DR36-B1006 led to a significantly lower efficiency (47.6% ± 17.8%) than pYL-*kasO*p^∗^-*Fn*Cas12a2-*actII-orf4*-DR36 (100%) (Supplementary Figure 11). Thus, the oop terminator of the crRNA array was responsible for the higher editing efficiency of the *Fn*Cas12a system. In conclusion, the two systems described in the present study provide researchers with additional choices for manipulation of different *Streptomyces* strains.

Genome editing and transcriptional repression in *Streptomyces* with the CRISPR-Cas12a system have also recently been reported by Li et al. (2018). Those researchers obtained an editing efficiency for single-gene deletion with a CRISPR-Cas12a system of 95%, which was lower than that obtained with our *Fn*Cas12a2 system (100%). However, the editing efficiencies of pYL-*ermE*p^∗^-*Fn*Cas12a1*-actII*-*orf4*-*DR19* and pYL-*ermE*p^∗^-*Fn*Cas12a2-*actII*-*orf4*-*DR19* were lower than those of their CRISPR-*Fn*Cas12a system carrying Cas12a controlled by the same promoter, *ermE*p^∗^. Several factors may explain this discrepancy. First, the promoter controlling the crRNA cassette in the CRISPR-Cas12a system described by Lei Li et al. was *kasO*p^∗^, which is stronger than the *gapdh*(EL) (Shao et al., 2013; Myronovskyi and Luzhetskyy, 2016) promoter used in our study. The high crRNA expression level may have increased the editing efficiency. Second, the strains were different. The authors of the previous study used *S. hygroscopicus* SIPI-KF and *E. coli* S17-1. *E. coli* S17-1 contains a chromosomally integrated derivative of RP4, which stimulates the integration of DNA from the donor strain into the recipient genome (Simon et al., 1983; Voeykova et al., 1998; Kieser et al., 2000). Thus, the status of the *Fn*Cas12a system in *Streptomyces* may be influenced by different donor strains. Importantly, *Fn*Cas12a was successfully applied in the current study to accurately delete large chromosomal DNA fragments ranging from 24 to 128 kb with efficiencies ranging from 25% to 92.9% ± 7.2% based on HDR, and the editing efficiency obtained using HDR was much higher than that obtained using NHEJ repair (10%, 27.6 kb). Thus, HDR was more suitable than NHEJ for deleting large DNA fragments. Moreover, the *Fn*Cas12a3 system has an expanded PAM recognition ability, which might provide increased opportunities for researchers to conduct precise genome editing. This system will be particularly useful for generating insertions and site mutations, as it facilitates selection of PAMs in the limited DNA sequence regions in *Streptomyces* strains with a high GC content. Therefore, the three *Fn*Cas12a systems we developed are versatile tools for precise genome editing in different *Streptomyces* strains.

In summary, the engineering tools developed in the present study are applicable for biosynthetic pathway reconstruction, metabolic engineering, and chassis cell construction in different *Streptomyces* strains. These tools will also be beneficial for natural product discovery and overproduction.

## Data Availability Statement

All datasets generated for this study are included in the article/Supplementary Material.

## Author Contributions

JZha, HL, SL, and YL designed the experiments. JZha, DZ, and JZhu performed the experiments. DZ, JZha, and YL wrote the manuscript. All authors contributed to the article and approved the submitted version.

## Conflict of Interest

The authors declare that the research was conducted in the absence of any commercial or financial relationships that could be construed as a potential conflict of interest.

## Footnotes

1 https://www.idtdna.com/scitools/Applications/Real Time PCR/
