# Supplementary material for: Efficient Multiplex Genome Editing in Streptomyces via Engineered CRISPR-Cas12a Systems
Source: Front Bioeng Biotechnol. 2020 Jun 30;8:726. doi: 10.3389/fbioe.2020.00726 (PMC7338789; doi:10.3389/fbioe.2020.00726)
Supplement: Supplementary file 1 [file Image_1.pdf]

Supplementary Material

1 Supplementary Figures

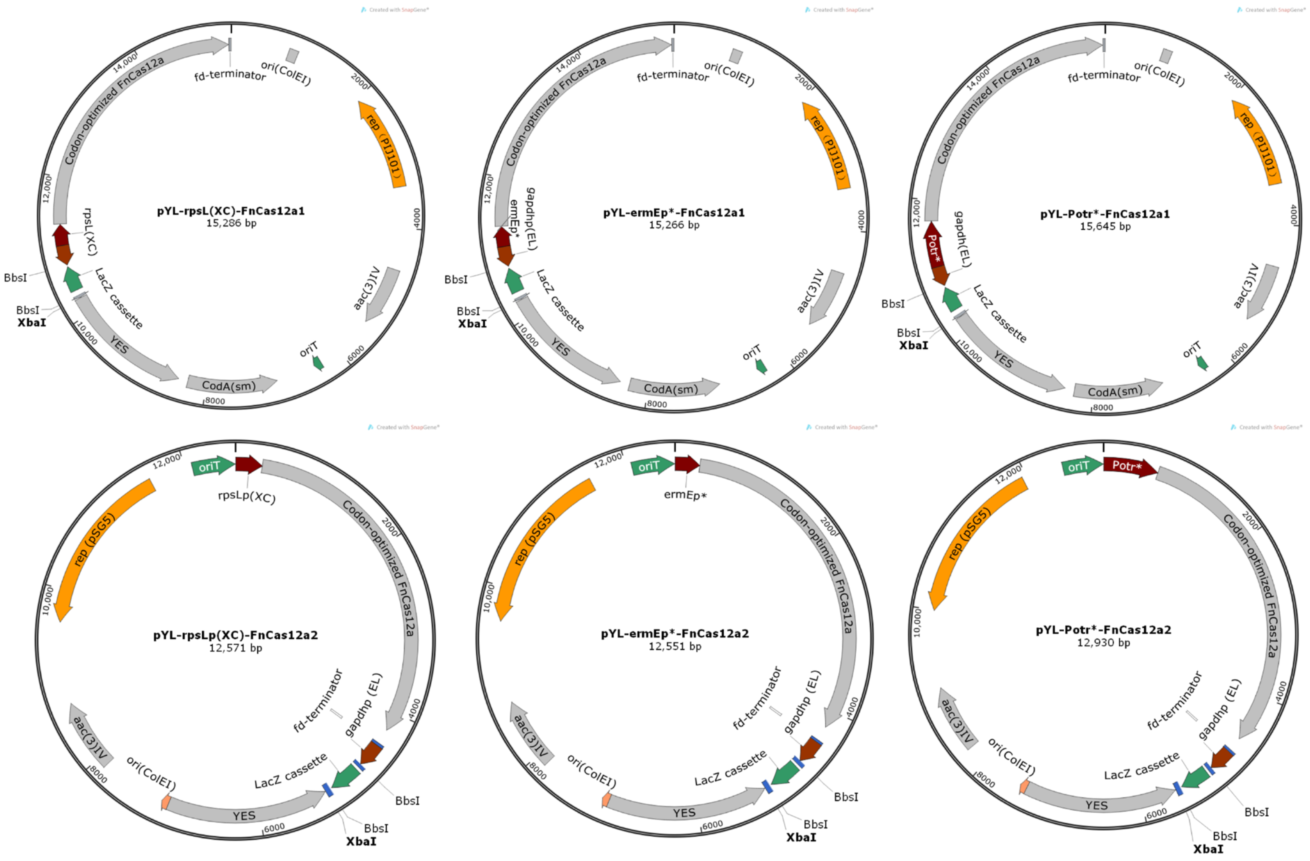

Supplementary Figure 1. Plasmids carrying *Fn*Cas12a with different promoters.

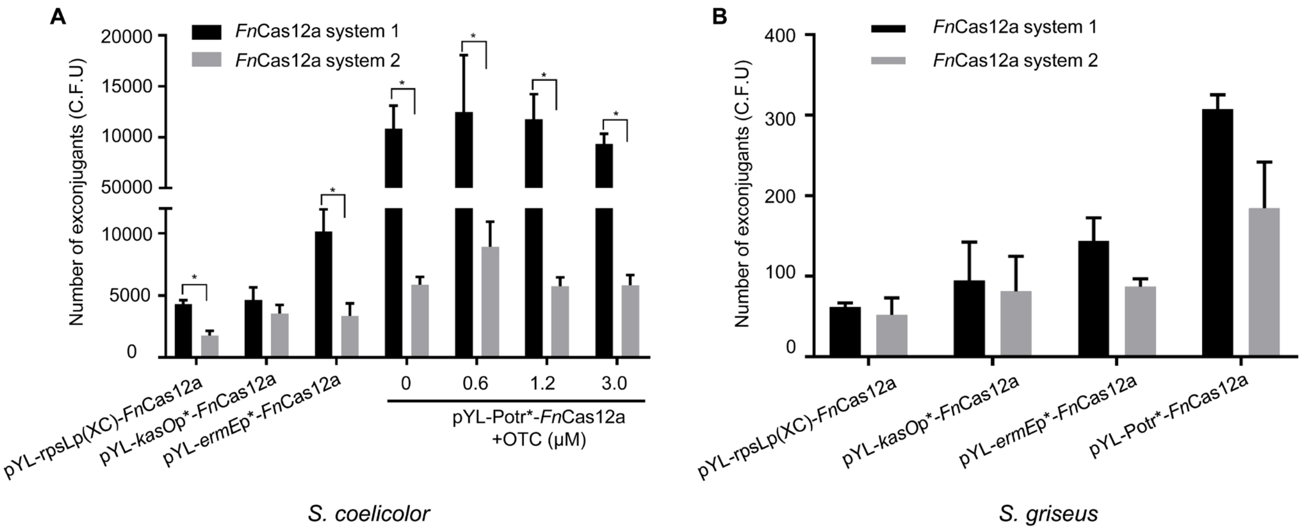

Supplementary Figure 2. Comparison of transformation frequencies between the *Fn*Cas12a1 system and the *Fn*Cas12a2 system. * represents a statistically significant difference. The experiments were performed in triplicate.

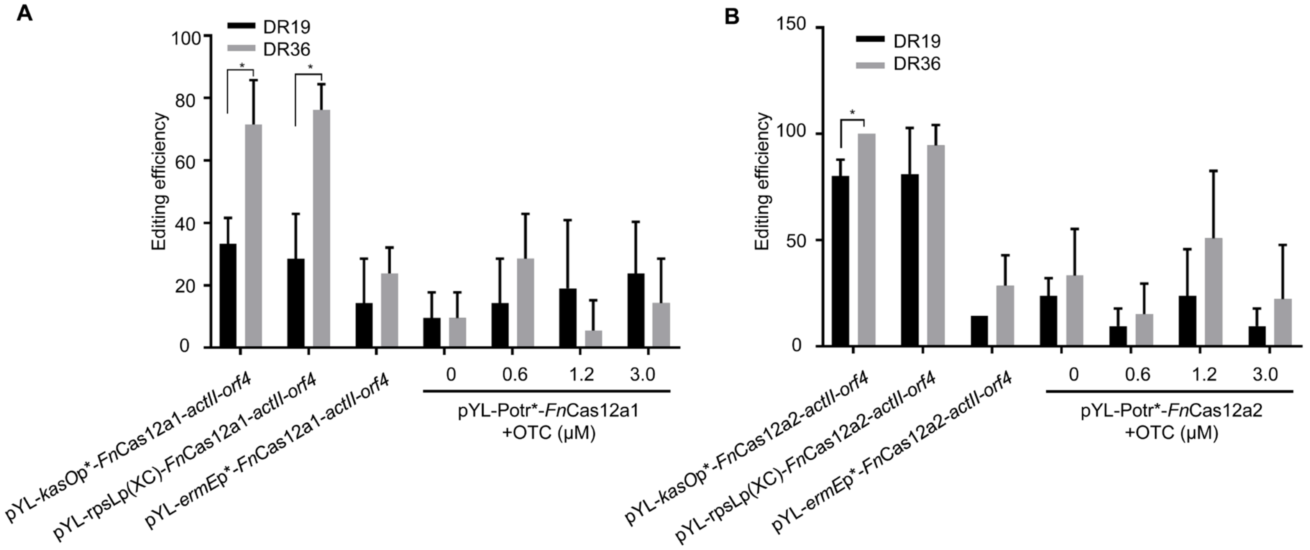

Supplementary Figure 3. Comparison of the editing efficiencies of *Fn*Cas12a systems paired with 19-nt DRs and *Fn*Cas12a systems paired with 36-nt DRs. * represents a statistically significant difference. The experiments were performed in triplicate.

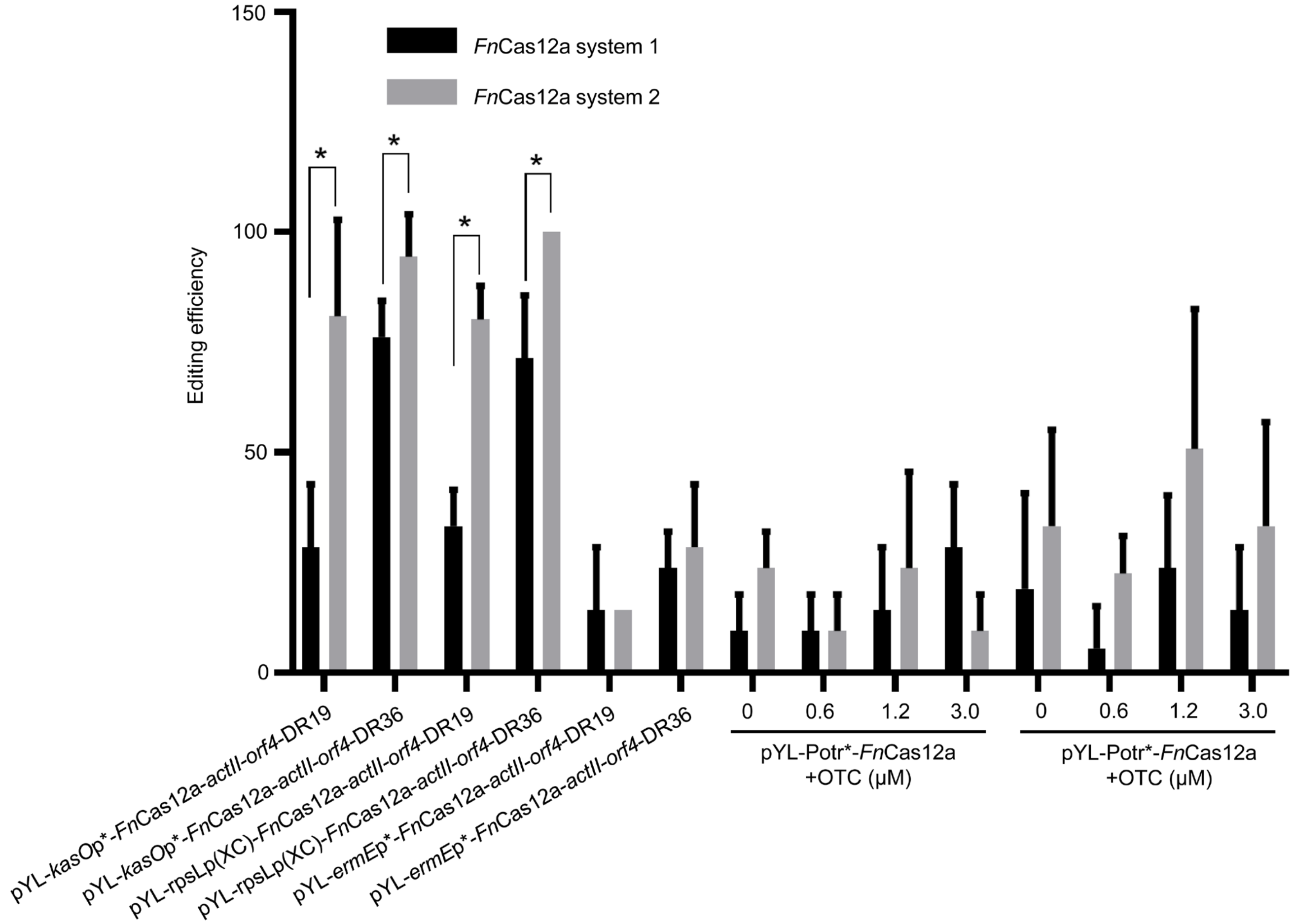

Supplementary Figure 4. Comparison of the editing efficiencies between the *Fn*Cas12a1 system and the *Fn*Cas12a2 system. * represents a statistically significant difference. The experiments were performed in triplicate.

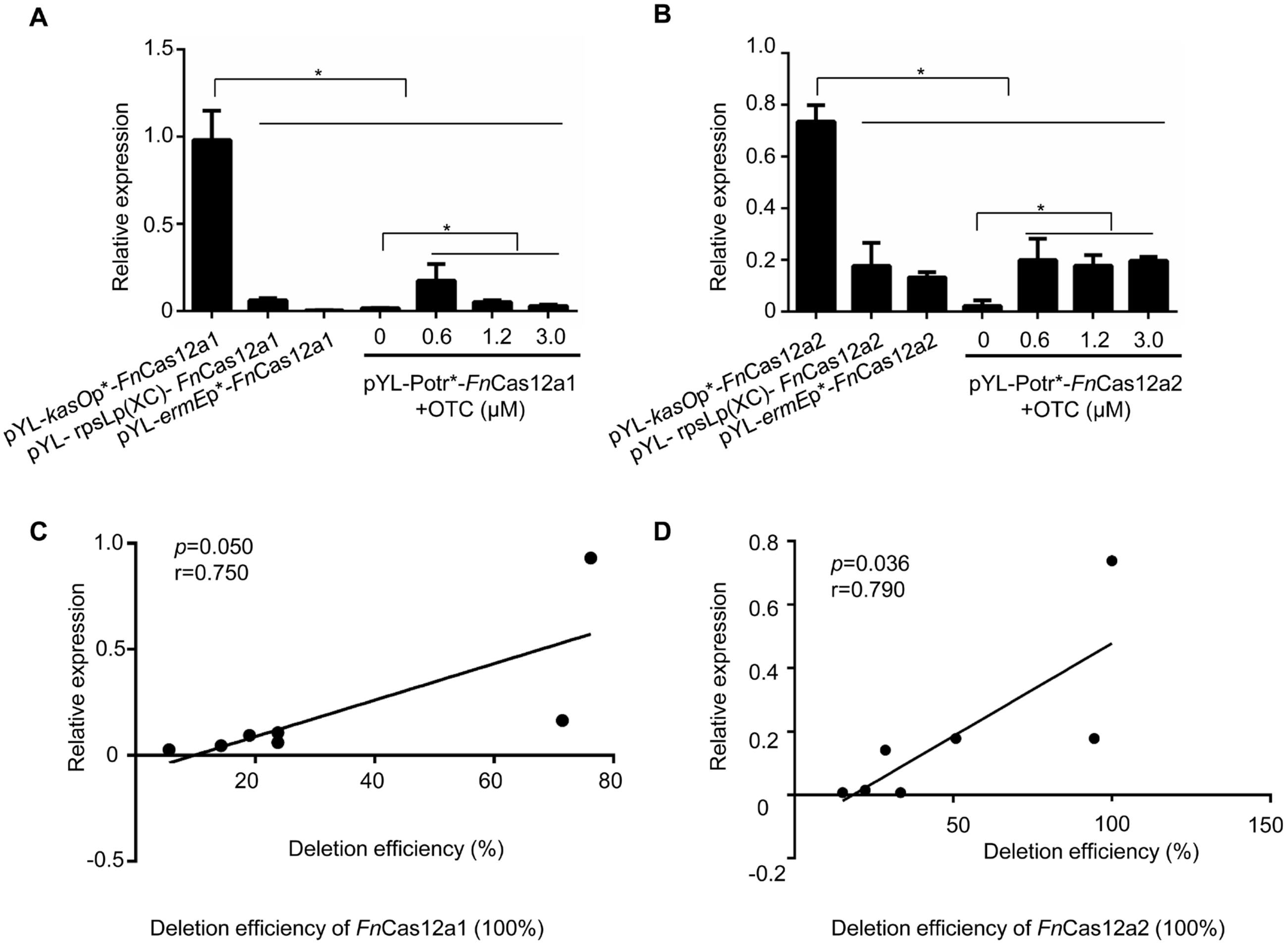

Supplementary Figure 5. Correlations between transcription levels and editing efficiencies based on the two *Fn*Cas12a systems. (A) - (B) Quantitative analyses of the transcription levels of *Fn*Cas12a1 and *Fn*Cas12a2 driven by rpsLp(XC), *kasO*p*, *ermE*p* and Potr* in response to OTC in *S. coelicolor*. (C) - (D) Correlation between transcription levels of *Fn*Cas12a1 and *Fn*Cas12a2 systems and their corresponding editing efficiencies. The experiments were performed in triplicate. $p < 0.05$ represents a statistical significance.

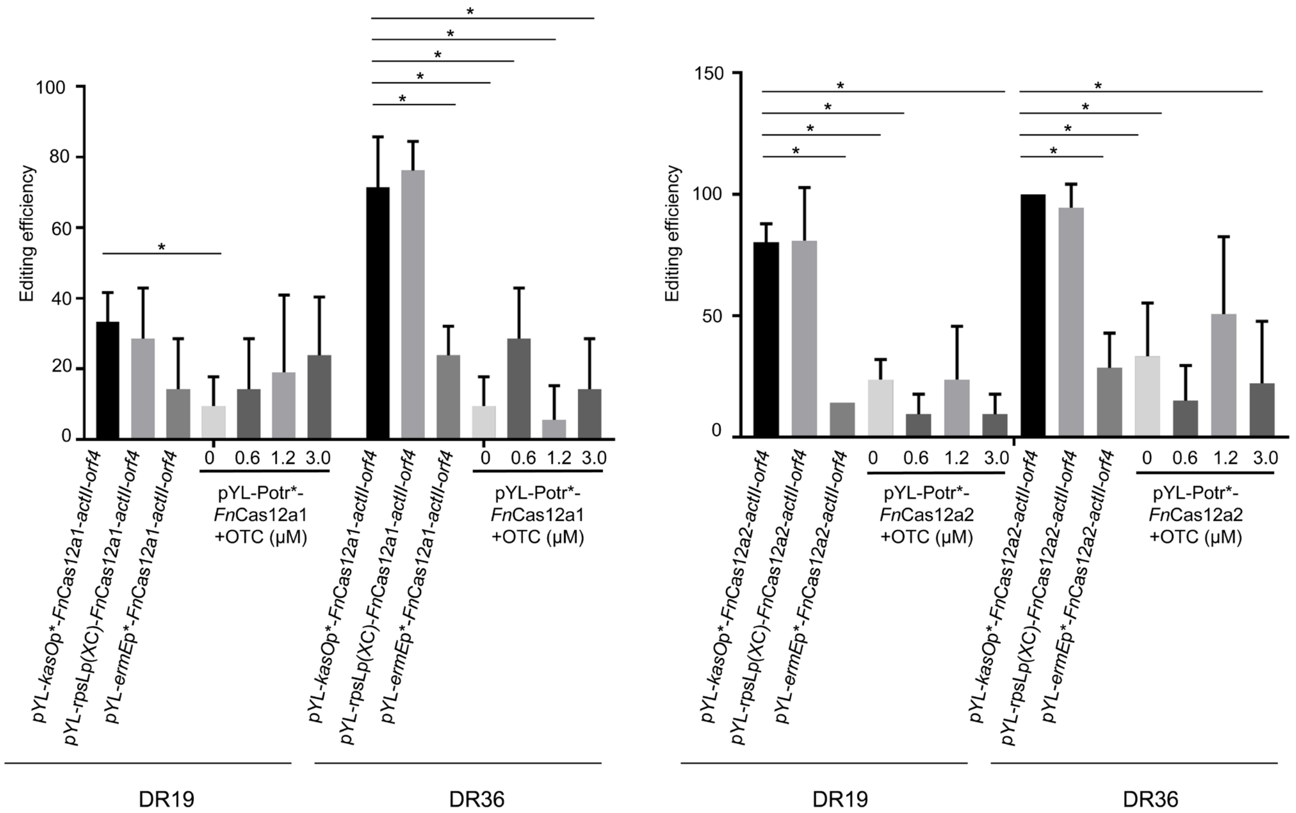

Supplementary Figure 6. Graph of the editing efficiencies of the *Fn*Cas12a1 and *Fn*Cas12a2 systems. * represents a statistically significant difference. The experiments were performed in triplicate.

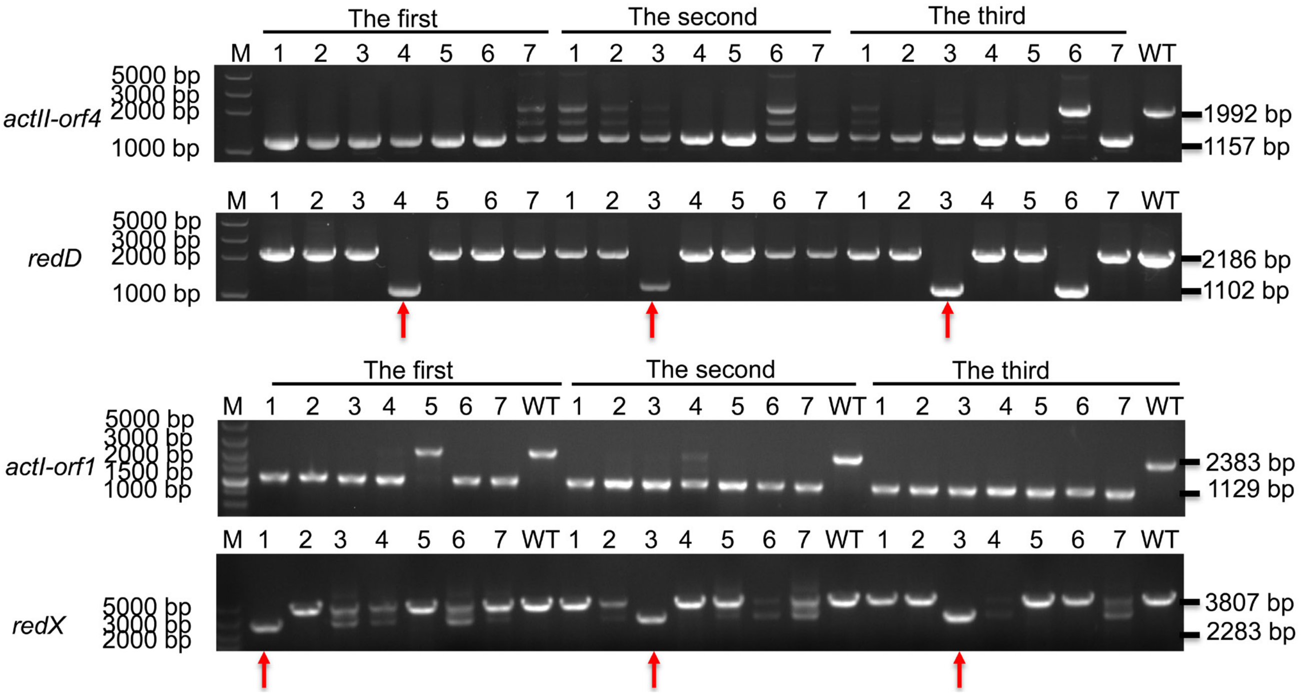

Supplementary Figure 7. Evaluation of double deletions via the *Fn*Cas12a2 system. The double-deletion mutants are indicated by red arrows. The experiments were performed in triplicate.

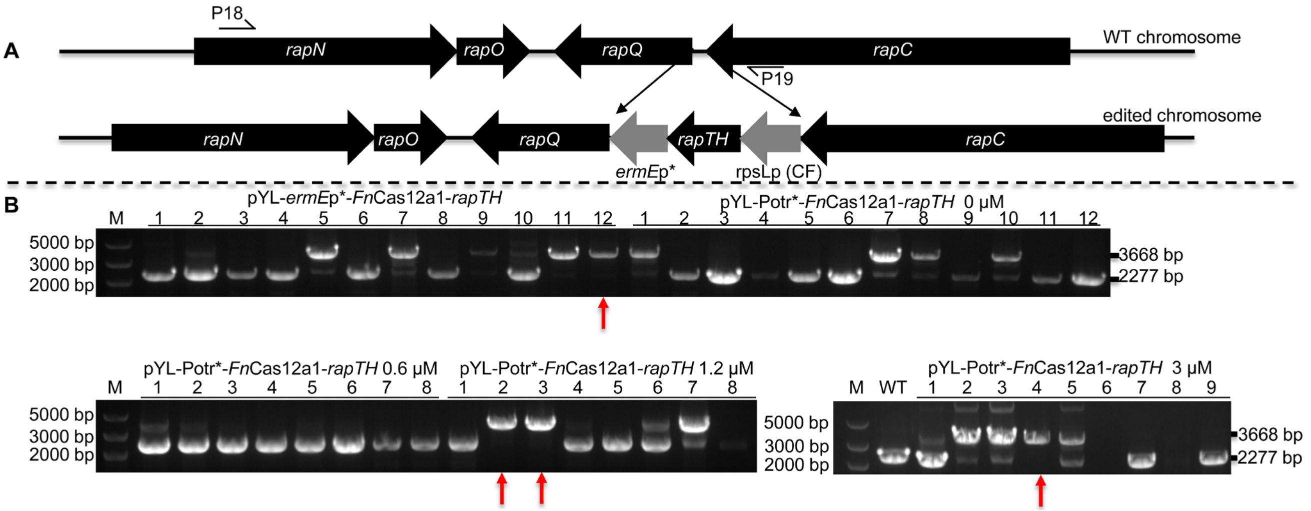

Supplementary Figure 8. Evaluation of a single insertion in *Streptomyces hygroscopicus* NRRL 5491. (A) Graphic representation of the recombination process. The *ermE*p* promoter, *RapTH* gene and rpsLp (CF) promoter were inserted into the site between *RapQ* and *RapC*. (B) PCR evaluation of the insertions conducted by pYL-*ermE*p*-*Fn*Cas12a1-*RapTH* and pYL-Potr*-*Fn*Cas12a1-*RapTH*. For the strains harboring successful insertions, the primers P18 and P19 generated a 3668-bp amplicon. In contrast, for the wild-type strains, the primers P18 and P19 generated a 2277-bp amplicon. Only one out of twelve strains exhibited successful insertion when using pYL-*ermE*p*-*Fn*Cas12a1-*RapTH*. The pYL-Potr*-*Fn*Cas12a1-*RapTH* plasmid resulted in a higher insertion efficiency when treating with 1.2 μm OTC. The successfully edited strains were indicated by red arrows. The experiments were performed in triplicate.

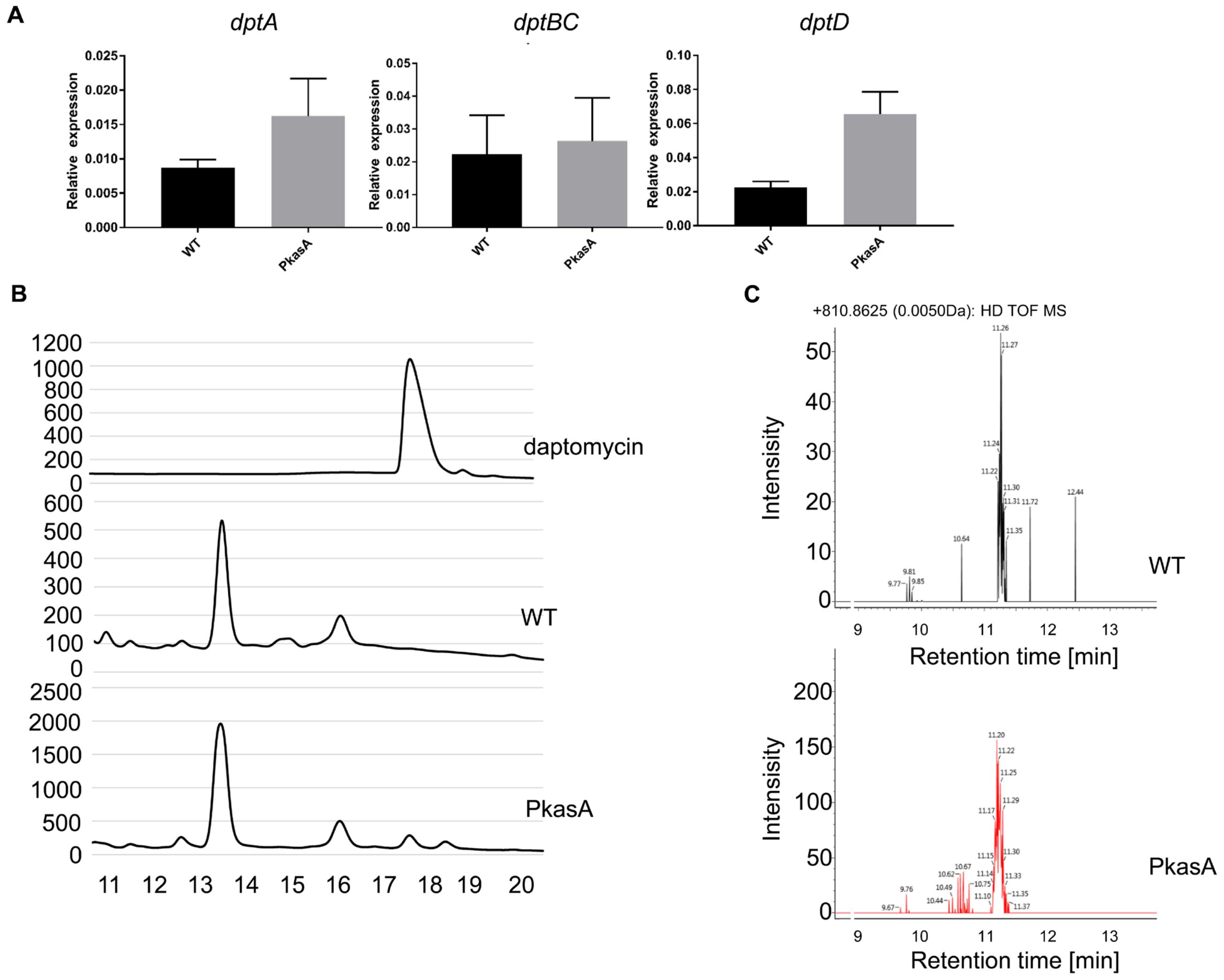

Supplementary Figure 9. Evaluation of daptomycin gene cluster activation. (A) Transcription analysis of daptomycin biosynthetic genes followed by the *kasO*p* promoter. (B) HPLC analysis of the daptomycin production. (C) Identification of daptomycin by MS, m/z: 810.8625. WT: wild-type, PkasA: *kasO*p* was introduced in front of the NRPS gene in the daptomycin biosynthetic gene cluster in *S. roseosporus*. The experiments were performed in triplicate.

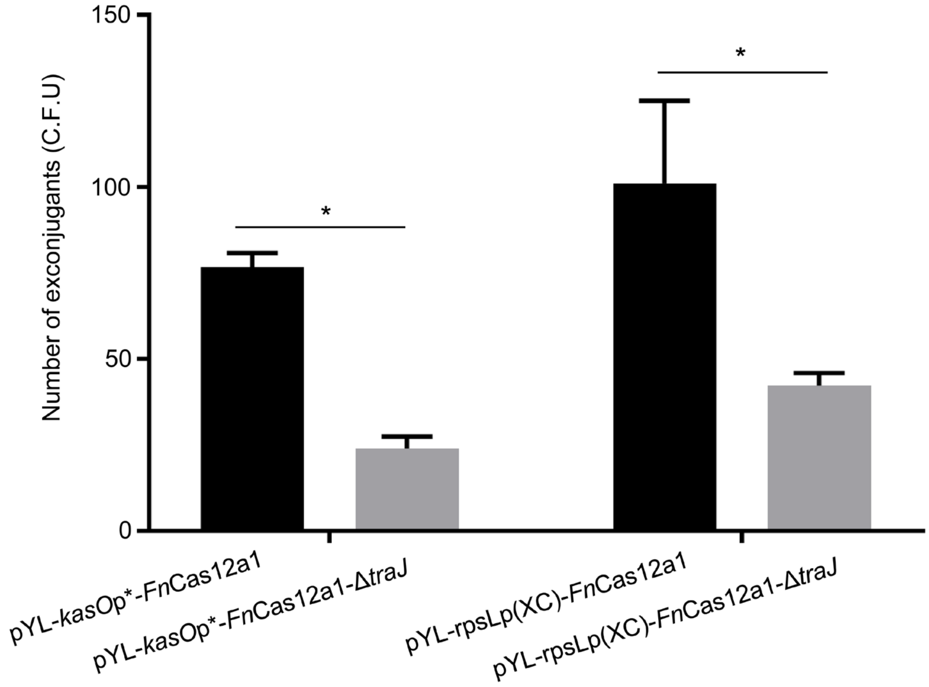

Supplementary Figure 10. Transformation frequencies of pYL-*kasO*p*-*Fn*Cas12a1-Δ*traJ* and pYL-rpsLp(XC)-*Fn*Cas12a1-Δ*traJ*. pYL-*kasO*p*-*Fn*Cas12a1-Δ*traJ: traJ* gene was deleted from pYL-*kasO*p*-*Fn*Cas12a1. pYL-rpsLp(XC)-*Fn*Cas12a1-Δ*traJ*: *traJ* gene was deleted from pYL-rpsLp(XC)-*Fn*Cas12a1. * represents a statistically significant difference. The experiments were performed in triplicate.

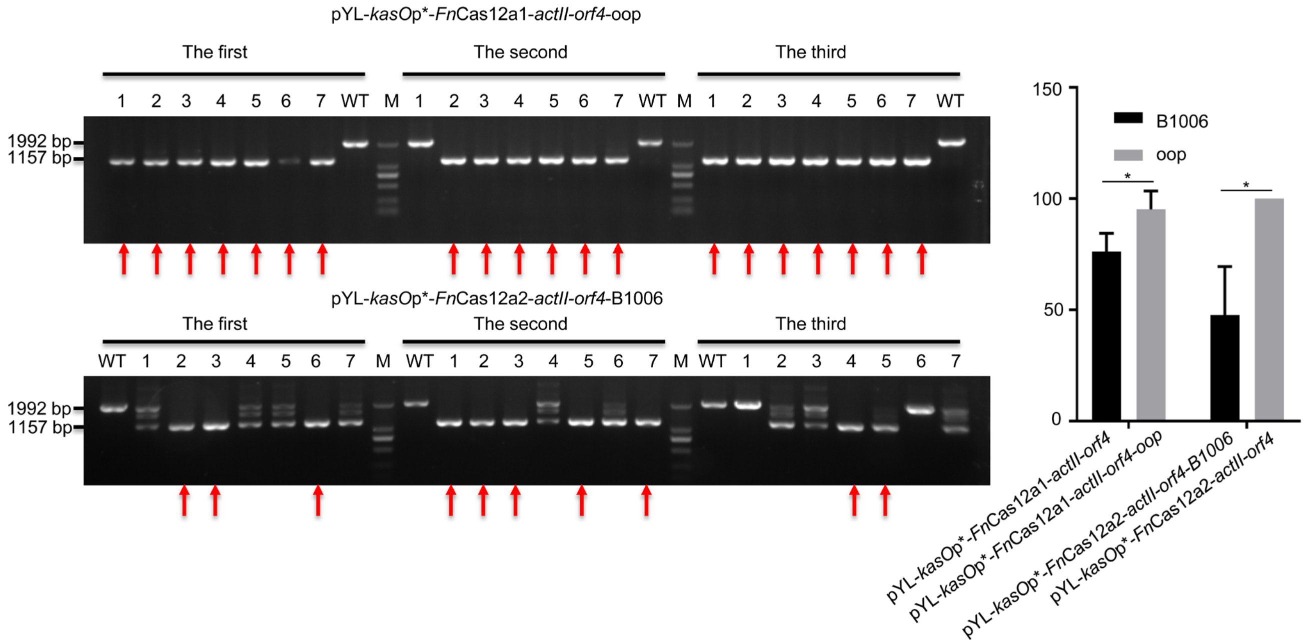

Supplementary Figure 11. Evaluation of the *actII-orf4* deletion resulted by pYL-*kasO*p*-*Fn*Cas12a1-*actII-orf4*-oop and pYL-*kasO*p*-*Fn*Cas12a2-*actII-orf4*-B1006. pYL-*kasO*p*-*Fn*Cas12a1-*actII-orf4*-oop: the terminator (B1006) of crRNA in pYL-*kasO*p*-*Fn*Cas12a1-*actII-orf4* was replaced with oop. pYL-*kasO*p*-*Fn*Cas12a2-*actII-orf4*-B1006: the terminator of crRNA (oop) in pYL-*kasO*p*-*Fn*Cas12a1-*actII-orf4* was replaced with B1006. M stands for the DNA marker; and the sizes from top to bottom are: 2000, 1000, 750, 500, 250, 100 bp. * represents a statistically significant difference. The experiments were performed in triplicate.
