# Supplementary material for: Efficient Multiplex Genome Editing in Streptomyces via Engineered CRISPR-Cas12a Systems
Source: Front Bioeng Biotechnol. 2020 Jun 30;8:726. doi: 10.3389/fbioe.2020.00726 (PMC7338789; doi:10.3389/fbioe.2020.00726)
Supplement: Supplementary file 2 [file Table_1.docx]

Supplementary Material

# Supplementary Tables

**Supplementary Table 1**. List of primers used in this study

| Description | Primer name | Primer sequence |
| --- | --- | --- |
| *Fn*Cas12a1 system construction | 2653-1-For | ACTGAGAATTCAATTAAAGGCTCCTTTTGGAGCCTTTTTTTCTGTCAGACCAAGTTTAC |
|  | 2653-1-Rev | GCAACAACATGAATGGTCATCGGTTTCCGTGTTTCGTAAAGTCTGGAAACGCGGAAGTC |
|  | 2653-2-For | CGAAACACGGAAACCGATGACCATTCATGTTGTTGCTCAGGTCGCAGACGTTTTGCAGC |
|  | 2653-2-Rev | GCTCGCGGACCTCATGGACGCCCTCCAGGGCACCCGGAAAACGCCGGACAGCCCCCGGC |
|  | 2653-3-For | GTCGCTGGTAGGCGCCCGGCCGCCGGGGGCTGTCCGGCGTTTTCCGGGTGCCCTGGAGG |
|  | 2653-3-Rev | TTATTTTTATAGCACGTGATGAAAAGGACCCAGGTGGCACTTTTCGTACCCGGGGATCC |
|  | 2653-yeast-For | GATTCTGGTCTAGCTAGAGTCGACTAGAGGATCCCCGGGTACGAAAAGTGCCACCTGGG |
|  | 2653-yeast-Rev | CGAAAAAAAAACCCCGCCCCTGACAGGGCGGGGTTTTTTTTTCTAGAGTGAGTTTAGTATACATGC |
|  | *lacZ*-For | TCTAGAAAAAAAAACCCCGCCCTGTCAGGGGCGGGGTTTTTTTTTCGGTCTTCACCGGT |
|  | LacZ-15-Rev | ACGGGGCCGGGCACCGTCGGGCCGGCGGCGCGGAACTAGTGCTGCTCCTTCGGTCGGAC |
|  | SA-15-For | TGCCCGTAGACGCACGTCCGACCGAAGGAGCAGCACTAGTTCCGCGCCGCCGGCCCGAC |
|  | SA-15-Rev | AGTACTTGTTCACAAATTCTTGGTAGATGGACATGCTAGCGGGTTCCTCCTCATGAGTC |
|  | Cas12a-15-For | TGCAGTGAACAAGTGGACTCATGAGGAGGAACCCGCTAGCATGTCCATCTACCAAGAAT |
|  | Cas12a-Rev | CTGACAGAAAAAAAGGCTCCAAAAGGAGCCTTTAATTGAATTCTCAGTTATTGCGGTTC |
|  | *ermE*p*-For | GACGCACGTCCGACCGAAGGAGCAGCGGTACCAGCCCGACCCGAG |
|  | *ermE*p*-Rev | CAAATTCTTGGTAGATGGACATGCTAGCGTGGTGTCCTACCAACCGG |
|  | *kasO*p*-For | GACGCACGTCCGACCGAAGGAGCAGCTGTTCACATTCGAACGGTC |
|  | *kasO*p*-Rev | CAAATTCTTGGTAGATGGACATGCTAGCAACTCCCCCAGTCCTGCAC |
|  | *kasO*p*-1-For | TGTTCACATTCGAACGGTCTCTGCTTTGACAACATGCTGTGCGGTGTTGTAAAGTCGTG |
|  | *kasO*p*-1-Rev | AACTCCCCCAGTCCTGCACGCTGTCGTATTCTCCTGGCCACGACTTTACAACACCGCAC |
|  | Potr*-For | GACGCACGTCCGACCGAAGGAGCAGCTCAGGCGGACTGCCGCCCG |
|  | Potr*-Rev | CAAATTCTTGGTAGATGGACATGCTAGCGGTTCCTCACTCTCCTGGC |
|  | rpsLp(XC)-For | GACGCACGTCCGACCGAAGGAGCAGCGCCCTGCAGGCGGAAGTCA |
|  | rpsLp(XC)-BbsI-Rev | CAAATTCTTGGTAGATGGACATGCTAGCTACGTCTCCGTCGTCTACT |
| *Fn*Cas12a2 system construction | 2-*lacZ* For | GTATCTGAAAGGGGATACGCAAGTCTTCCGGTGGAAAGCGGGCAGTG |
|  | 2-*lacZ* Rev | CGTTCTGAACAAATCCAGATGGAGTATGTCTTCTCAGCCGCTACAGG |
|  | 2-gapdh For | GTTCGTCCAGAACCGCAATAACTGAGAATTCAGATCTACGCGTTC |
|  | 2-gapdh Rev | CACTGCCCGCTTTCCACCGGAAGACTTGCGTATCCCCTTTCAGATAC |
|  | 2-Yes For | CCTGTAGCGGCTGAGAAGACATACTCCATCTGGATTTGTTCAG |
|  | 2-Yes Rev | CAAAGGATCTTCTTGAGATCC |
|  | 2-acc For | GCAAGCAGCAGATTACGCGC |
|  | 2-acc Rev | GAGGAGGAGATCACCGACGAC |
|  | c-pSG For | GATGACCGCGATGGCGAC |
|  | 2-pSG Rev | GACTTCCGCCTGCAGGGCAAGCTTCAGCTCGCGGACGTGCTCA |
|  | 2-rpsl For | TGAGCACGTCCGCGAGCTGAAGCTTGCCCTGCAGGCGGAAGTC |
|  | 2-rpsl Rev | ATTCTTGGTAGATGGACATGGATCCTACGTCTCCGTCGTCTACTC |
|  | 2-Cas12a For | GAGTAGACGACGGAGACGTAGGATCCATGTCCATCTACCAAGAAT |
|  | 2-Cas12a Rev | GAACGCGTAGATCTGAATTCTCAGTTATTGCGGTTCTG |
|  | 2-*kasO*p*-pCFc-For | TGCAGGACTGGGGGAGTTGGATCCATGTCCATCTACCAAG |
|  | 2-*kasO*p*-pCFc-Rev | TGCAGGACTGGGGGAGTTGGATCCATGTCCATCTACCAAG |
|  | 2-*ermE*p*-pCFc-For | CGTCGTGGACTATGAGCACGTCCGCGAGCTGAAGCTTGGTACCAGCCCGACCCGA |
|  | 2-*ermE*p*-pCFc-Rev | CTTGGTAGATGGACATGGATCCGTGGTGTCCTACCAACCGGCAC |
|  | 2-Potr*-pCFc-For | GCGTCGTGGACTATGAGCACGTCCGCGAGCTGAAGCTTTCAGGCGGACTGCCGCCCGGA |
|  | 2-Potr*-pCFc-Rev | CTTGGTAGATGGACATGGATCCGGTTCCTCACTCTCCTG |
| *ActII-orf4* deletion using *Fn*Cas12a1 system with the 23-nt spacer followed by a 19-nt or a 36-nt direct repeat | 1-*actII*4-19-sp-For | TTTTCGCGTCGATACGGAGCTGCATTCATCTACAACAGTAGAAATT |
|  | 1-*actII*4-19-sp-Rev | ACGCAATTTCTACTGTTGTAGATGAATGCAGCTCCGTATCGACGCG |
|  | 1-*actII*4-36-sp-For | TTTTCGCGTCGATACGGAGCTGCATTCATCTACAACAGTAGAAATTATTTAAAGTTCTTAGAC |
|  | 1-*actII*4-36-sp-Rev | ACGCGTCTAAGAACTTTAAATAATTTCTACTGTTGTAGATGAATGCAGCTCCGTATCGACGCG |
|  | 1- *actII*4-KL-For | CTGTATTATAAGTAAATGCATGTATACTAAACTCACTC |
|  | 1- *actII*4-KL-Rev | CTACTGACGCCGGCTGCGCCCCCGTCGAGATTC |
|  | 1- *actII*4-KR-For | GACGGGGGCGCAGCCGGCGTCAGTAGTTCCCCAG |
|  | 1- *actII*4-KR-Rev | ACCCCGCCCCTGACAGGGCGGGGT |
|  | 1-Cas12a-SP-check-For | GGCTGGGAAGCATATTTG |
|  | 1-Cas12a-SP-check-Rev | CTGCTCCTTCGGTCGGAC |
|  | P1 | GTGGGCGAGGAGATCCTGAC |
|  | P2 | ATGGTGGCGTACCTGATC |
| *ActII-orf4* deletion using *Fn*Cas12a2 system with the 23-nt spacer followed by a 19-nt or a 36-nt direct repeat | 2- *actII*4-19-sp-For | ACGCAATTTCTACTGTTGTAGATGAATGCAGCTCCGTATCGACGCG |
|  | 2- *actII*4-19-sp-Rev | GAGTCGCGTCGATACGGAGCTGCATTCATCTACAACAGTAGAAATT |
|  | 2- *actII*4-36-sp-For | ACGCGTCTAAGAACTTTAAATAATTTCTACTGTTGTAGATGAATGCAGCTCCGTATCGACGCG |
|  | 2- *actII*4-36-sp-Rev | GAGTCGCGTCGATACGGAGCTGCATTCATCTACAACAGTAGAAATTATTTAAAGTTCTTAGAC |
|  | 2- *actII*4-KL-For | TGCCGCCGGGCGTTTTTTATCTAGAGGTGAACCCATGGTCGTC |
|  | 2- *actII*4-KR-Rev | GACCCAGGTGGCACTTTTCGTCTAGAACGACTCTGCGCTTCAATC |
|  | 2-spacer-2kb check For | GTGAATGGCCTGTTC |
|  | 2-spacer-2kb check Rev | GATCCCCCTAGAGTC |
| ACT deletion using pYL-*kasO*p*-*Fn*Cas12a2 plasmid | ACT-sp-For 1 | ACGCGTCTAAGAACTTTAAATAATTTCTACTGTTGTAGATGAATGCAGCTCCGTATCGACGCG |
|  | ACT-sp-Rev 1 | GAGTCGCGTCGATACGGAGCTGCATTCATCTACAACAGTAGAAATTATTTAAAGTTCTTAGAC |
|  | ACT-KL For | CAGAACGCTCGGTTGCCGCCGGGCGTTTTTTATCTAGACTCTCGAACACGGCGGTCAC |
|  | ACT-KL Rev | CACCCACATGGTGGAGGAGACCGGTGAACGGACGCTGACAG |
|  | ACT-KR For | CTGTCAGCGTCCGTTCACCGGTCTCCTCCACCATGTGGGTG |
|  | ACT-KR Rev | TGATGAAAAGGACCCAGGTGGCACTTTTCGTCTAGAGAACGTCCGCCTGGTCGAGAC |
|  | P3 | CTGTGCTGCTTTTCGCGCCTG |
|  | P4 | CGCTTCCCACCGGCCTGTAC |
|  | P5 | GACGCGGTCACGTTCTGGGAC |
| CDA knockout using pYL-*kasO*p*-*Fn*Cas12a2 plasmid | CDA-spacer1-For | TTTTTGTTCAGATCGGTGGTCGGACCCATCTACAACAGTAGAAATTATTTAAAGTTCTTAGAC |
|  | CDA-spacer1-Rev | ACGCGTCTAAGAACTTTAAATAATTTCTACTGTTGTAGATGGGTCCGACCACCGATCTGAACA |
|  | CDA-spacer2-For | TTTTGCGGGAAGTTCCTGGGCGAGGTCATCTACAACAGTAGAAATTATTTAAAGTTCTTAGAC |
|  | CDA-spacer2-Rev | ACGCGTCTAAGAACTTTAAATAATTTCTACTGTTGTAGATGACCTCGCCCAGGAACTTCCCGC |
|  | CDA-spacer3-For | TTTTCTGGAGTTGAGCGGATAGTTCTCATCTACAACAGTAGAAATTATTTAAAGTTCTTAGAC |
|  | CDA-spacer3-Rev | ACGCGTCTAAGAACTTTAAATAATTTCTACTGTTGTAGATGAGAACTATCCGCTCAACTCCAG |
|  | CDA-2KL-For | CATATCATCAATACTTGTCACATGAGAGTACTGATCTACAAGGTCGACG |
|  | CDA-2KL-Rev | GAGGTGAACGCGGTCGTACTGCCGAAGGTAAG |
|  | CDA-2KR-For | GCAGTACGACCGCGTTCACCTCTGGGGCTC |
|  | CDA-2KR-Rev | CTTCAGGCATCAAATTTCAGTGGCCTCGCCCTAGACGACCGCGAG |
|  | CDA-HR-CM-For | GTGCGAGTATCTGAAAGGGGATACGCGTCTAAGAACTTTAAATAATTTCTACTG |
|  | CDA-HR-CM-Rev | GAAAAGGACCCAGGTGGCACTTTTCGTCTAGAGCATATCATCAATACTTGT |
|  | PCM2-CDA-For1 | ACGCTCGGTTGCCGCCGGGCGTTTTTTAGCATATCATCAATACTTGTC |
|  | PCM2-CDA-Rev1 | ACGTGATGAAAAGGACCCAGGTGGCACTTTTCGTCTAGAGCTGTGCTTTATGAATC |
|  | P6 | GACCACGGAGTACACGCTGA |
|  | P7 | CGGCATCCATCTCGAACTCAC |
|  | P8 | CTCCGACACCACCGAACAG |
| DAP knockout using pYL-*kasO*p*-*Fn*Cas12a2 and pCRISPomyces-2 plasmids | DAP-spacer-For1 | ACGCGTCTAAGAACTTTAAATAATTTCTACTGTTGTAGATAGTGCGCCGACCGTCCCGTGCTC |
|  | DAP-spacer-Rev1 | AAACGAGCACGGGACGGTCGGCGCACTATCTACAACAGTAGAAATTATTTAAAGTTCTTAGAC |
|  | DAP spacer For2 | ACGCGTCTAAGAACTTTAAATAATTTCTACTGTTGTAGATaactggggcatcagtgcctggac |
|  | DAP spacer Rev2 | AAACGTCCAGGCACTGATGCCCCAGTTATCTACAACAGTAGAAATTATTTAAAGTTCTTAGAC |
|  | DAP-KL-For | CAGAACGCTCGGTTGCCGCCGGGCGTTTTTTATCTAGAGTCGATGCCTTCCTGGAGCTG |
|  | DAP-KL-Rev | GCATTCCCCACGGCTTCACGCGCCGGGGAAGTCCTCTGTC |
|  | DAP-KR-For | GACAGAGGACTTCCCCGGCGCGTGAAGCCGTGGGGAATGC |
|  | DAP-KR-Rev | CGTGATGAAAAGGACCCAGGTGGCACTTTTCGTCTAGACACCAGCGCCCACCCGAAC |
|  | P9 | CAGGAGGGTGGGGAGCAGATC |
|  | P10 | CATTCGGTCGCGGCACATGC |
|  | P11 | TCGTGCTCGCCCGAATACAG |
| *RapTH* insertion | Cas12a-*RapTH*-SP-For | TTTTCCGTACTTGGCACATCGGCTGGGATCTACAACAGTAGAAATTATTTAAAGTTCTTAGAC |
|  | Cas12a-*RapTH*-SP-Rev | ACGCGTCTAAGAACTTTAAATAATTTCTACTGTTGTAGATCCCAGCCGATGTGCCAAGTACGG |
|  | Cas12a-*RapTH*-SP-Rev | GAGTCCGTACTTGGCACATCGGCTGGGATCTACAACAGTAGAAATTATTTAAAGTTCTTAGAC |
|  | TH-KL-For | AAAAACTGTATTATAAGTAAATGCATGTATACTAAACTCACTCTAGACCGAAATGGAGC |
|  | TH-KL-Rev | AGCTCATCGAGCTCATCGCCAAGAAGCTCGCGGGCTGAGTGATGTTGGAATTGGGTAAC |
|  | TH-KR-For | TAAATCTGAACTTCAGGCGGTTACCCAATTCCAACATCACTCAGCCCGCGAGCTTCTTG |
|  | TH-KR-Rev | AAAAAAAAACCCCGCCCCTGACAGGGCGGGGTTTTTTTTTCTAGACAGCGTGATCAAG |
|  | TH-HR-For | CTGTATTATAAGTAAATGCATGTATACTAAACTCACTCTAGACAGCGTGATCAAGATGG |
|  | TH-HR-Rev | ACCCCGCCCCTGACAGGGCGGGGTTTTTTTTCCGAAATGGAGCGACTCGAG |
|  | P18 | GACCGATCAGGGTGAGAC |
|  | P19 | CAGCTCTCGGCAAAATGGAG |
|  | Cas12a1-RapTH-SP For | TTTTCCGTACTTGGCACATCGGCTGGGATCTACAACAGTAGAAATTATTTAAAGTTCTTAGAC |
|  | Cas12a1-RapTH-SP Rev/ Cas12a2-RapTH-SP For | ACGCGTCTAAGAACTTTAAATAATTTCTACTGTTGTAGATCCCAGCCGATGTGCCAAGTACGG |
|  | Cas12a2-RapTH-SP Rev | GAGTCCGTACTTGGCACATCGGCTGGGATCTACAACAGTAGAAATTATTTAAAGTTCTTAGAC |
| RT-PCR analyses of *Fn*Cas12a in *Streptomyces* | *hrdB*-T For | GAGCCGCTCCCGGTTCCAC |
|  | *hrdB*-T Rev | GCATGCGGGCTCCTCACTC |
|  | RT *Fn*Cas12a For | CAACACGGCCATTCTGTTTATT |
|  | RT *Fn*Cas12a Rev | CCTTCTTCGGGTTGTCCTTATT |
|  | RT *hrdB* For | TTGATGACCTCGACCATGTG |
|  | RT *hrdB* Rev | GCGGTGGAGAAGTTCGACTA |
| Insertion of *kasO*p* in front of *dptA* gene using pYL-*kasO*p*-*Fn*Cas12a3 plasmid | Cas9-pYL-*kasO*p*-For | AGCGTGCAGGACTGGGGGAGTTATGGACAAGAAGTACAGCATCG |
|  | Cas9-pYL-*kasO*p*-Rev | CGGGGAACGCGTAGATCTGAATTC |
|  | Dapt-ccg-sp-For | ACGCGTCTAAGAACTTTAAATAATTTCTACTGTTGTAGATCGAGAGATGGACATGCAGTCGCA |
|  | Dapt-ccg-sp-Rev | GAGTTGCGACTGCATGTCCATCTCTCGATCTACAACAGTAGAAATTATTTAAAGTTCTTAGAC |
|  | Dapt-atc-sp-For | ACGCGTCTAAGAACTTTAAATAATTTCTACTGTTGTAGATCGCGAGAGATGGACATGCAGTCG |
|  | Dapt-atc-sp-Rev | GAGTCGACTGCATGTCCATCTCTCGCGATCTACAACAGTAGAAATTATTTAAAGTTCTTAGAC |
|  | Dapt-cca-sp-For | ACGCGTCTAAGAACTTTAAATAATTTCTACTGTTGTAGATTCTCTCGCGGATCCCCTCAGGTG |
|  | Dapt-cca-sp-Rev | GAGTCACCTGAGGGGATCCGCGAGAGAATCTACAACAGTAGAAATTATTTAAAGTTCTTAGAC |
|  | Dapt-KL-For | AACGCTCGGTTGCCGCCGGGCGTTTTTTATCTAGAAGGACAACTCGTGGACCACG |
|  | Dapt-KL-Rev | GACTGGGGGAGTTATGGACATGCAGTCGCAGC |
|  | Dapt- *kasO*p*-For | CTGCATGTCCATAACTCCCCCAGTCCTGCAC |
|  | Dapt- *kasO*p*-Rev | CGACCGCACCTGATGTTCACATTCGAACGGTC |
|  | Dapt-KR-For | GAATGTGAACATCAGGTGCGGTCGGCCAAC |
|  | Dapt-KR-Rev | TGATGAAAAGGACCCAGGTGGCACTTTTCGTCTAGAGAGGCCACCCTGTTCGTG |
|  | Dapt-p-check-F | GAATTCCCCGTCGCGCCG |
|  | pCFa-YES-Rev | AGCACGTGATGAAAAGGAC |
|  | Dapt-kas-Check-For | GCCGTAGGAGTTGATGTGG |
|  | Dapt-kas-check-innner-rev | CGCCGAGTTCGTGAACGAG |
|  | Dapt-kas-check-Rev | TGCGGTGTTGTAAAGTCGTG |
| *Fn*Cas12a3 system construction | *Fn*Cas12a-N607R-for | GACTTTAGCGcgCGGCTGGGATAAAAACAAG |
|  | *Fn*Cas12a-N607R-rev | ATCCCAGCCGcgCGCTAAAGTCGAGTTCTC |
|  | 2653-*Fn*Cas12a-pam-k613v-Fn617r-for | GATAAAAACGTGGAGCCCGACCGCACGGCCATTCTGTTTAT |
|  | 2653-*Fn*Cas12a-pam-k613v-n617r-rev | GAATGGCCGTGCGGTCGGGCTCCACGTTTTTATCCCAGCCGT |
|  | 2653-*Fn*Cas12a-Bd616n-for-new- | ACGTGGAGCCCAACCGCACGGCCATTCTGTTTAT |
|  | 2653-*Fn*Cas12a-Bd616n-rev-new | TGGCCGTGCGGTTGGGCTCCACGTTTTTATC |
|  | 2653-*Fn*Cas12a-pam-k660r-for | GAAGATTGTGTATAGACTGCTGCCGGGGGC |
|  | 2653-*Fn*Cas12a-pam-k660r-rev | GCCCCCGGCAGCAGTCTATACACAATCTTCTTGTA |
|  | 2653-*Fn*Cas12a-pam-k671r-for | CAAAATGCTCCCGCGAGTTTTTTTCTCGGCG |
|  | 2653-*Fn*Cas12a-pam-k671r-rev | GAGAAAAAAACTCGCGGGAGCATTTTGTTG |
|  | 2653-*Fn*Cas12a-pam-k180s-for | CGACGTACTTCTCGGGCTTCCATGAGAACCG |
|  | 2653-*Fn*Cas12a-pam-k180s-rev | CATGGAAGCCCGAGAAGTACGTCGTCCAGC |
|  | seq-*Fn*Cas12a-1 | CACGAGCACACTGGGGCAC |
|  | seq-*Fn*Cas12a-2 | AGCGGAAGAGCTGACGTTC |
|  | seq-*Fn*Cas12a-3 | CAATTTAGCCCAGATCTCCA |
|  | seq-*Fn*Cas12a-4 | AGGTTATAAGCTCACCTTC |
| *actII-orf4* and *redD* double deletion using pYL-*kasO*p*-*Fn*Cas12a2 plasmid | AD-P1 | GAGACATCTTTGAAGACTTACGCGTCTAAGAACTTTAAATAATTTCTACTGTTGTAG |
|  | AD-P2 | ACCGCGTCGATACGGAGCTGCATTCATCTACAACAGTAGAAATTATTTAAAG |
|  | AD-P3 | GAGACATCTTTGAAGACTTACGCGTC |
|  | AD-P4 | ACCGCGTCGATACGGAGCTG |
|  | AD-P5 | CAGCTCCGTATCGACGCGGTCTAAGAACTTTAAATAATTTCTACTGTTGTAGATCTGAG |
|  | AD-P6 | CGTGAAGAAGACATGAGTGCGCCCTGCGAGACGAGTCTCAGATCTACAACAGTAG |
|  | AD-P7 | CAGCTCCGTATCGACGCGGT |
|  | AD-P8 | GCCACGTGAAGAAGACATGAGTGC |
|  | A-KL-For | GTTCAGAACGCTCGGTTGC |
|  | A-KR-Rev | GAGGTCCTGCTCATCACGACTCTGCGCTTCAATC |
|  | D-KL-For | GAAGCGCAGAGTCGTGATGAGCAGGACCTCGTG |
|  | D-KR-Rev | AGCACGTGATGAAAAGGACCCAGGTGGCACTTTTCGGACAGCGAGTAGCGGAAG |
|  | AD-P1-1 | TCTTTGAAGACTTACGCGTCTAAGAACTTTAAATAATTTCTACTGTTGTAGATGAATGC |
|  | AD-P2-1 | ACCTGACTTCCGCCTGCAGGGCCGCGTCGATACGGAGCTGCATTCATCTACAACAGTAG |
|  | AD-P5-1 | CGAGTAGACGACGGAGACGTAGTCTAAGAACTTTAAATAATTTCTACTGTTGTAGATCT |
|  | AD-P6-1 | CGTGAAGAAGACATGAGTGCGCCCTGCGAGACGAGTCTCAGATCTACAACAGTAGAAAT |
|  | T7-*actII*-Rev | AGACCCGTTTAGAGGCCCCAAGGGGTTATGCTACGCGTCGATACGGAGCTG |
|  | P12 | ACGCGTTCGACCGAGATG |
|  | P13 | TGTGGCTGTGTCGTTGTC |
| *actI-orf1* and *redX* double deletion using pYL-*kasO*p*-*Fn*Cas12a2 plasmid | AX-P1-1 | TCTTTGAAGACTTACGCGTCTAAGAACTTTAAATAATTTCTACTGTTGTAGATGGATTG |
|  | AX-P2-1 | ACCTGACTTCCGCCTGCAGGGCATGACGACTCTGCGCTTCAATCCATCTACAACAGTAG |
|  | AX-P4 | ACATGACGACTCTGCGCTTC |
|  | AX-P5-1 | CGAGTAGACGACGGAGACGTAGTCTAAGAACTTTAAATAATTTCTACTGTTGTAGATCC |
|  | AX-P6-1 | CGTGAAGAAGACATGAGTTGCCTTCCTCGATCAACAGGTGGATCTACAACAGTAGAAAT |
|  | AX-P7 | GAAGCGCAGAGTCGTCATGT |
|  | AX-P8 | GCCACGTGAAGAAGACATGAGTTG |
|  | rpsLp(XC)-For | GCCCTGCAGGCGGAAGTCAG |
|  | rpsLp(XC)-Rev | TACGTCTCCGTCGTCTACTCG |
|  | T7-*actI*-Rev | AGACCCGTTTAGAGGCCCCAAGGGGTTATGCTAATGACGACTCTGCGCTTC |
|  | T7-rpsLp(XC)-For | CTTGGGGCCTCTAAACGGGTCTTGAGGGGTTTTTTGGCCCTGCAGGCGGAAGTCAG |
|  | *actI*-KL-For | CAGAACGCTCGGTTGCCGCCGGGCGTTTTTTATCTAGAACGGTGAGAAGGTGCTCGTG |
|  | *actI*-KL-Rev | GAGATCGCACTCGTCCAGCGGCACCCCATCT |
|  | *actI*-KR-For | GTGCCGCTGGACGAGTGCGATCTCGACTACGTTC |
|  | *actI*-KR-Rev | ACCATGAGCAGTTGCAACGTCCTCGGGACCGGTC |
|  | *redX* -KL-For | ACCGGTCCCGAGGACGTTGCAACTGCTCATGGTGGAG |
|  | *redX* -KL-Rev | GTCCTCCAGGAGCACGTGGCATAC |
|  | *redX* -KR-For | GTATGCCACGTGCTCCTGGAGGACGCAACGCGATGAACTTC |
|  | *redX* -KR-Rev | AGCACGTGATGAAAAGGACCCAGGTGGCACTTTTCGCGTCCAGTCCGAGTTGTAC |
|  | P14 | CTGGAATCGTATCGGAATCTCC |
|  | P15 | TGAGTACGGACCGCAGCTTC |
|  | P16 | ACATCGAGGTCGACGTGGCAC |
|  | P17 | TCGTTGGTGCCGGAGAAGATC |
| Site mutations rpsL (nt262 A>G, nt264G>A, nt267C>T) using pYL-*kasO*p*-*Fn*Cas12a3 plasmid | rpsL-SP-For | ACGCGTCTAAGAACTTTAAATAATTTCTACTGTTGTAGATTGTGAAGGACCTGCCGGGTGTTC |
|  | rpsL-SP-Rev | GAGTGAACACCCGGCAGGTCCTTCACAATCTACAACAGTAGAAATTATTTAAAGTTCTTAGAC |
|  | rpsL-KL-For | GTTCAGAACGCTCGGTTGCCGCCGGGCGTTTTTTACAGGTGACGTACGAGCGCGCGCC |
|  | rpsL-KL-Rev | AACACCCGGCAGATCTTCCACACGGCCGCCGCGCACGA |
|  | rpsL-KR-For | CCGTGTGGAAGATCTGCCGGGTGTTCGCTAC |
|  | rpsL-KR-Rev | AGCACGTGATGAAAAGGACCCAGGTGGCACTTTTCGAGCTTGTTCACGAAGCAGATGC |
|  | rpsL-check-For | GTCTACTACCGGCTCATG |
|  | rpsL-check-Rev | CAGTCTTCTCTCGGTCTC |
| Deletion of partial sequences of *traJ* gene from *Fn*Cas12a1 system | 1-Δ*traJ*-For | TGAGCACGTCCGCGAGCTGTCAGCGCTTGTAGTCGATGGCCTC |
|  | 1-Δ*traJ*-Rev | AGGCCATCGACTACAAGCGCTGACAGCTCGCGGACGTGCTCATAGTC |
| Replace B1006 terminator of crRNA in *Fn*Cas12a1 with oop terminator | 1*-actII-orf4*-DR36-oop For | CTATGATCGGGGCGTTCCTGC |
|  | 1-*actII-orf4*-DR36-oop Rev | GCAAAACTAAAAAACTGTATTATAAGTAAATGCATGTATACTAAACTCACACGACTCTGCGCTTCAATC |
| Replace oop terminator of crRNA in *Fn*Cas12a2 system with B1006 terminator | 2-*actII-orf4*-DR36-oop For | CTATGATCGGGGCGTTCCTGC |
|  | 2-*actII-orf4*-DR36-oop Rev | ATAATTATTTTTATAGCACGTGATGAAAAGGACCCAGGTGGCACTTTTCGGGTGAACCCATGGTCGTC |

**Supplementary Table 2**. Different elements among the *Fn*Cas12a1, *Fn*Cas12a2 and *Fn*Cas12a3 systems

| Descriptions | *Fn*Cas12a1 | *Fn*Cas12a2 | *Fn*Cas12a3 |
| --- | --- | --- | --- |
| Element for transfer | *oriT*, *traJ* | *oriT,* partial sequences of *traJ* | *oriT*, partial sequences of *traJ* |
| Selection marker | *aac(3)IV*, *codA(sm)* | *aac(3)IV*, *pSG5* | *aac(3)IV*, *pSG5* |
| Replication origin in *Streptomyces* | *rep* (pIJ101) | *rep* (pSG5) | *rep* (pSG5) |
| Terminator of crRNA cassette | B1006 terminator | oop terminator | oop terminator |
| *Fn*Cas12a | wide-type *Fn*Cas12a | wide-type *Fn*Cas12a | *Fn*Cas12a mutation EP16 |

**Supplementary Table 3**. List of plasmids used in this study

| Plasmids | Description | References |
| --- | --- | --- |
| pCRISPomyces-2 | CRISPR/Cas system carrying *Sp*Cas9 controlled by the rpsLp(XC) promoter | (Cobb et al., 2015) |
| pWHU2653 | CRISPR/Cas9-*CodA(sm)* combined system carrying *Sp*Cas9 controlled by the *aac(3)IVp* promoter | (Zeng et al., 2015) |
| pYL-rpsLp(XC)-*Fn*Cas12a1 | *Fn*Cas12a1 system carrying Cas12a controlled by the rpsLp(XC) promoter | This study |
| pYL-*kasO*p*-*Fn*Cas12a1 | *Fn*Cas12a1 system carrying Cas12a controlled by the *kasO*p* promoter | This study |
| pYL-*ermE*p*-*Fn*Cas12a1 | *Fn*Cas12a1 system carrying Cas12a controlled by the *ermE*p* promoter | This study |
| pYL-Potr*-*Fn*Cas12a1 | *Fn*Cas12a1 system carrying Cas12a controlled by the Potr* system | This study |
| pYL-rpsLp(XC)-*Fn*Cas12a2 | *Fn*Cas12a2 system carrying Cas12a controlled by the rpsLp(XC) promoter | This study |
| pYL-*kasO*p*-*Fn*Cas12a2 | *Fn*Cas12a2 system carrying Cas12a controlled by the *kasO*p* promoter | This study |
| pYL-*ermE*p*-*Fn*Cas12a2 | *Fn*Cas12a2 system carrying Cas12a controlled by the *ermE*p* promoter | This study |
| pYL-Potr*-*Fn*Cas12a2 | *Fn*Cas12a2 system carrying Cas12a controlled by the Potr* system | This study |
| pYL-rpsLp(XC)-*Fn*Cas12a1-*actII*-*orf4*-DR19 | pYL*-*rpsLp(XC)*-Fn*Cas12a1 plasmid carrying the 23-nt spacer targeting *actII*-*orf4* and a 19-nt DR sequence | This study |
| pYL-rpsLp(XC)-*Fn*Cas12a1-*actII*-*orf4*-DR36 | pYL*-*rpsLp(XC)*-Fn*Cas12a1 plasmid carrying the 23-nt spacer targeting *actII*-*orf4* and a 36-nt DR sequence | This study |
| pYL-*kasO*p*-*Fn*Cas12a1-*actII*-*orf4*-DR19 | pYL*-kasO*p**-Fn*Cas12a1 plasmid carrying the 23-nt spacer targeting *actII*-*orf4* and a 19-nt DR sequence | This study |
| pYL-*kasO*p*-*Fn*Cas12a1-*actII*-*orf4*-DR36 | pYL*-kasO*p**-Fn*Cas12a1 plasmid carrying the 23-nt spacer targeting *actII*-*orf4* and a 36-nt DR sequence | This study |
| pYL-*ermE*p*-*Fn*Cas12a1-*actII*-*orf4*-DR19 | pYL*-ermE*p**-Fn*Cas12a1 plasmid carrying the 23-nt spacer targeting *actII*-*orf4* and a 19-nt DR sequence | This study |
| pYL-*ermE*p*-*Fn*Cas12a1-*actII*-*orf4*-DR36 | pYL*-ermE*p**-Fn*Cas12a1 plasmid carrying the 23-nt spacer targeting *actII*-*orf4* and a 36-nt DR sequence | This study |
| pYL-Potr*-*Fn*Cas12a1-*actII*-*orf4*-DR19 | pYL-Potr**-Fn*Cas12a1 plasmid carrying the 23-nt spacer targeting *actII*-*orf4* and a 19-nt DR sequence | This study |
| pYL-Potr*-*Fn*Cas12a1-*actII*-*orf4*-DR36 | pYL-Potr**-Fn*Cas12a1 plasmid carrying the 23-nt spacer targeting *actII*-*orf4* and a 36-nt DR sequence | This study |
| pYL-rpsLp(XC)-*Fn*Cas12a2-*actII*-*orf4*-DR19 | pYL*-*rpsLp(XC)*-Fn*Cas12a2 plasmid carrying the 23-nt spacer targeting *actII*-*orf4* and a 19-nt DR sequence | This study |
| pYL-rpsLp(XC)-*Fn*Cas12a2-*actII*-*orf4*-DR36 | pYL*-*rpsLp(XC)*-Fn*Cas12a2 plasmid carrying the 23-nt spacer targeting *actII*-*orf4* and a 36-nt DR sequence | This study |
| pYL-*kasO*p*-*Fn*Cas12a2-*actII*-*orf4*-DR19 | pYL*-kasO*p**-Fn*Cas12a2 plasmid carrying the 23-nt spacer targeting *actII*-*orf4* and a 19-nt DR sequence | This study |
| pYL-*kasO*p*-*Fn*Cas12a2-*actII*-*orf4*-DR36 | pYL*-kasO*p**-Fn*Cas12a2 plasmid carrying the 23-nt spacer targeting *actII*-*orf4* and a 36-nt DR sequence | This study |
| pYL-*ermE*p*-*Fn*Cas12a2-*actII*-*orf4*-DR19 | pYL*-ermE*p**-Fn*Cas12a2 plasmid carrying the 23-nt spacer targeting *actII*-*orf4* and a 19-nt DR sequence | This study |
| pYL-*ermE*p*-*Fn*Cas12a2-*actII*-*orf4*-DR36 | pYL*-ermE*p**-Fn*Cas12a2 plasmid carrying the 23-nt spacer targeting *actII*-*orf4* and a 36-nt DR sequence | This study |
| pYL-Potr*-*Fn*Cas12a2-*actII*-*orf4*-DR19 | pYL-Potr**-Fn*Cas12a2 plasmid carrying the 23-nt spacer targeting *actII*-*orf4* and a 19-nt DR sequence | This study |
| pYL-Potr*-*Fn*Cas12a2-*actII*-*orf4*-DR36 | pYL*-*Potr**-Fn*Cas12a2 plasmid carrying the 23-nt spacer targeting *actII*-*orf4* and a 36-nt DR sequence | This study |
| pYL-*kasO*p*-*Fn*Cas12a2-ACT | pYL*-kasO*p**-Fn*Cas12a2 plasmid carrying the 23-nt spacer targeting ACT gene cluster and a 36-nt DR sequence | This study |
| pCRISPomyces-2-DAP | pCRISPomyces-2 plasmid carrying the 20-nt spacer targeting DAP gene cluster and an 81-nt gRNA sequence | This study |
| pYL-*kasO*p*-*Fn*Cas12a2-DAP | pYL-*kasO*p**-Fn*Cas12a2 plasmid carrying the 23-nt spacer targeting DAP gene cluster and a 36-nt DR sequence | This study |
| pYL*-ermE*p*-*Fn*Cas12a1-*rapTH*-DR36 | pYL-*ermE*p**-Fn*Cas12a1 plasmid carrying the 23-nt spacer targeting *rapTH* and a 36-nt DR sequence | This study |
| pYL-Potr*-*Fn*Cas12a1-*rapTH*-DR36 | pYL-Potr**-Fn*Cas12a1 plasmid carrying the 23-nt spacer targeting *RapTH* gene and a 36-nt DR sequence | This study |
| pYL-*kasO*p*-*Fn*Cas12a1-*rapTH*-DR36 | pYL-*kasO*p**-Fn*Cas12a1 plasmid carrying the 23-nt spacer targeting *RapTH* gene and a 36-nt DR sequence | This study |
| pYL-*kasO*p*-*Fn*Cas12a3 | pYL-*kasO*p*-*Fn*Cas12a3 plasmid carrying *Fn*Cas12a mutant EP16 (N607R/K613V/N617R/K180S/K660R/D616N) | This study |
| pYL-*kasOp** -*Fn*Cas12a3-DAP (CCG) | pYL-*kasO*p*-*Fn*Cas12a3 carrying a 36-nt DR with a spacer adjacent to CCG PAM | This study |
| pYL-*kasOp**-*Fn*Cas12a3-DAP(ATC) | pYL-*kasO*p*-*Fn*Cas12a3 carrying a 36-nt DR with a spacer adjacent to ATC PAM | This study |
| pYL-*kasO*p*-*Fn*Cas12a3-DAP(CCA) | pYL-*kasO*p*-*Fn*Cas12a3 carrying a 36-nt DR with a spacer adjacent to CCA PAM | This study |
| pYL-*kasO*p*-*Fn*Cas12a3-rpsL | This plasmid carrying the 23-nt spacer targeting *rpsL* gene and a 36-nt DR sequence | This study |
| pYL-*kasO*p*-*Fn*Cas12a2-*actII*-rpsLp(XC)-T7-*redD*-4kb | This plasmid carrying two 23-nt spacers targeting *actII-orf4* and *redD* respectively and a 36-nt DR sequences | This study |
| pYL-*kasO*p*-*Fn*Cas12a2-*actI*- rpsLp(XC)-T7-*redX*-4kb | This plasmid carrying two 23-nt spacers targeting *actI-orf1* and *redX* respectively and two 36-nt DR sequences | This study |
| pYL-*kasO*p*-FnCas12a1-Δ*traJ* | The partial sequences of *traJ* gene were deleted from pYL-rpsLp(XC)-*Fn*Cas12a1 | This study |
| pYL-rpsLp(XC)-FnCas12a1-Δ*traJ* | The partial sequences of *traJ* gene were deleted from pYL-*kasO*p*-*Fn*Cas12a1 | This study |

**Supplementary Table 4**. Deletion efficiencies in the *actII*-*orf4* gene deletion experiments by using two *Fn*Cas12a systems in the presence of OTC

| Plasmids | OTC (μM) | Deletion efficiency |
| --- | --- | --- |
| pYL-Potr*-*Fn*Cas12a1-*actII*-*orf4*-DR19 | 0 | 9.5%±6.7% |
|  | 0.6 | 9.5%±6.7% |
|  | 1.2 | 14.3%±11.7% |
|  | 3.0 | 28.6%±11.7% |
| pYL-Potr*-*Fn*Cas12a1-*actII*-*orf4*-DR36 | 0 | 28.6%±14.3% |
|  | 0.6 | 5.6%±7.9% |
|  | 1.2 | 23.8%±13.5% |
|  | 3.0 | 14.3%±11.7% |
| pYL-Potr*-*Fn*Cas12a2-*actII*-*orf4*-DR19 | 0 | 23.8%±6.7% |
|  | 0.6 | 9.5%±6.7% |
|  | 1.2 | 35.7%±7.1% |
|  | 3.0 | 9.5%±6.7% |
| pYL-Potr*-*Fn*Cas12a2-*actII*-*orf4*-DR36 | 0 | 21.4%±7.1% |
|  | 0.6 | 15.1%±11.7% |
|  | 1.2 | 69.1%±2.4% |
|  | 3.0 | 22.2%±20.8% |

**Supplementary Table 5**. Efficiencies of multiplex and precise gene editing

| Targets | Plasmids | Efficiency |
| --- | --- | --- |
| ACT (deletion) | pYL-*kasO*p*-*Fn*Cas12a2-ACT | 92.9%±7.2% |
| CDA  (deletion) | pYL-*kasO*p*-*Fn*Cas12a2-CDA-sp1 | 55.6%±7.9% |
|  | pYL-*kasO*p*-*Fn*Cas12a2-CDA-sp2 | 18.8%±6.3% |
|  | pYL-*kasO*p*-*Fn*Cas12a2-CDA-sp3 | 25.0%±0.0% |
|  | pYL-*kasO*p*-*Fn*Cas12a2-CDA-2sp | 25.0%±0.0% |
| DAP  (deletion) | pYL-*kasO*p*-*Fn*Cas12a2-DAP-sp1 | 25.0%±0.0% |
|  | pYL-*kasO*p*-*Fn*Cas12a2-DAP-sp2 | 0.0%±0.0% |
| *RapTH* (insertion） | pYL-*ermE*p*-*Fn*Cas12a1-*RapTH*-DR36 | 8.3% |
|  | pYL-Potr*-*Fn*Cas12a1-*RapTH*-DR36 | 0.0% (0 μM OTC) |
|  |  | 0.0% (0.6 μM OTC) |
|  |  | 25.0% (1.2 μM OTC) |
|  |  | 8.3% (3.0 μM OTC) |
| DAP (insertion) | pYL-*kasO*p*-*Fn*Cas12a3-DAP(CCG) | 50.0%±12.5% |
|  | pYL-*kasO*p*-*Fn*Cas12a3-DAP(CCA) | 40.0%±17.4% |
|  | pYL-*kasO*p*-*Fn*Cas12a3-DAP(ATC) | 23.6%±8.6% |
| rpsL (mutation) | pYL-*kasO*p*-*Fn*Cas12a3-rpsL (CCG) | 12.5%±0.0% |
| *actII-orf4/redD* double deletion | pYL-*kasO*p*-*Fn*Cas12a2-*actII*-rpsLp(XC)-T7-*redD*-4kb | 14.3%±0.0% |
| *actI-orf1/redX* double deletion | pYL-*kasO*p*-*Fn*Cas12a2-*actI*-rpsLp(XC)-T7-*redX*-4kb | 14.3%±0.0% |
| *actII-orf4* | pYL-*kasO*p*-*Fn*Cas12a1-*actII-orf4*-DR36-oop | 95.2%±6.7% |
| *actII-orf4* | pYL-*kasO*p*-*Fn*Cas12a2-*actII-orf4*-DR36-B1006 | 47.6%±17.3% |

#

# References

Cobb, R.E., Wang, Y., and Zhao, H. (2015). High-efficiency multiplex genome editing of *Streptomyces* species using an engineered CRISPR/Cas system. *ACS Synth Biol* 4(6)**,** 723-728. doi: 10.1021/sb500351f.

Zeng, H., Wen, S., Xu, W., He, Z., Zhai, G., Liu, Y., et al. (2015). Highly efficient editing of the actinorhodin polyketide chain length factor gene in *Streptomyces coelicolor* M145 using CRISPR/Cas9-CodA(sm) combined system. *Appl Microbiol Biotechnol* 99(24)**,** 10575-10585. doi: 10.1007/s00253-015-6931-4.
